# Inorganic Phosphate Solubilization by Rhizosphere Bacterium *Paenibacillus sonchi*: Gene Expression and Physiological Functions

**DOI:** 10.3389/fmicb.2020.588605

**Published:** 2020-12-14

**Authors:** Luciana F. Brito, Marina Gil López, Lucas Straube, Luciane M. P. Passaglia, Volker F. Wendisch

**Affiliations:** ^1^Genetics of Prokaryotes, Faculty of Biology, Bielefeld University, Bielefeld, Germany; ^2^Center for Biotechnology, Bielefeld University, Bielefeld, Germany; ^3^Department of Genetics, Universidade Federal do Rio Grande do Sul, Porto Alegre, Brazil

**Keywords:** *Paenibacillus sonchi*, phosphate solubilization, PGPR – plant growth-promoting rhizobacteria, organic acids, osmoprotection, RNAseq

## Abstract

Due to the importance of phosphorus (P) in agriculture, crop inoculation with phosphate-solubilizing bacteria is a relevant subject of study. *Paenibacillus sonchi* genomovar Riograndensis SBR5 is a promising candidate for crop inoculation, as it can fix nitrogen and excrete ammonium at a remarkably high rate. However, its trait of phosphate solubilization (PS) has not yet been studied in detail. Here, differential gene expression and functional analyses were performed to characterize PS in this bacterium. SBR5 was cultivated with two distinct P sources: NaH_2_PO_4_ as soluble phosphate source (SPi) and hydroxyapatite as insoluble phosphate source (IPi). Total RNA of SBR5 cultivated in those two conditions was isolated and sequenced, and bacterial growth and product formation were monitored. In the IPi medium, the expression of 68 genes was upregulated, whereas 100 genes were downregulated. Among those, genes involved in carbon metabolism, including those coding for subunits of 2-oxoglutarate dehydrogenase, were identified. Quantitation of organic acids showed that the production of tricarboxylic acid cycle-derived organic acids was reduced in IPi condition, whereas acetate and gluconate were overproduced. Increased concentrations of proline, trehalose, and glycine betaine revealed active osmoprotection during growth in IPi. The cultivation with hydroxyapatite also caused the reduction in the motility of SBR5 cells as a response to Pi depletion at the beginning of its growth. SBR5 was able to solubilize hydroxyapatite, which suggests that this organism is a promising phosphate-solubilizing bacterium. Our findings are the initial step in the elucidation of the PS process in *P. sonchi* SBR5 and will be a valuable groundwork for further studies of this organism as a plant growth-promoting rhizobacterium.

## Introduction

Phosphorus (P) is a macronutrient required for plant growth. Plant roots can absorb P in the form of orthophosphates, either H_2_PO_4_^–^ or HPO_4_^2–^, but the concentration of these ions in the soil is in the micromolar range (Shin et al., 2004; Ai et al., 2009). This is due to the complex dynamics of P in the soil; this nutrient has a unique characteristic, which is its high fixation in soil. Mineral P can be, for example, found associated with the surface of iron or aluminum oxides in the soil, making it poorly soluble and therefore unavailable for plant nutrition (Shen et al., 2011). Those factors lead to the overuse of chemical P fertilizers and animal manure applied to agricultural land, which—although soil fertility and crop production are improved—caused severe environmental damage in the past decades, such as eutrophication of rivers and lakes, and the input of cadmium and radionuclides in the soil from contaminated fertilizers (Vance et al., 2003; Attallah et al., 2019; Li et al., 2020; Lee et al., 2020). Moreover, the input demand of P into croplands is expected to increase up to 86% by 2050 (Mogollón et al., 2018). It is also estimated an absolute shortage of P supply from industrial fertilizers in the future, which is aggravated by the depletion of the P present in agricultural soils caused by erosion (Alewell et al., 2020). Therefore, P is receiving more attention as a non-renewable resource (Cordell et al., 2009). Improvement of P acquisition and use by plants is critical for economic and environmental reasons. In this context, phosphate solubilization (PS) performed by organisms in the soil is an interesting target for study. PS ability is well characterized in mycorrhizal fungi (Delavaux et al., 2017) and phosphate-solubilizing bacteria (PSB), mostly associated with the plant rhizosphere (Rodríguez and Fraga, 1999). Endophytic and mycorrhizal fungal strains have a significant impact on plant P nutrition, being used by many agronomists as soil inoculants to enhance plant growth (Khan et al., 2010). Among rhizosphere fungi, the most common P-solubilizing genera are *Aspergillus*, *Penicillium*, and *Trichoderma* (Sharma et al., 2013; Wang et al., 2020). There is a substantial number of PSB genera widespread in the rhizosphere (Chen et al., 2006), of which some are well-established as plant growth-promoting rhizobacteria (PGPR), e.g., *Pseudomonas* (Parvaze et al., 2007), *Bacillus* (Chen et al., 2006), *Enterobacter* (Chung et al., 2005), and *Azotobacter* (Kumar et al., 2001). The use of PSB as inoculants leads to increased P uptake by the plant and improved crop yield (Nosratabad et al., 2017; Din et al., 2020; Magallon-Servin et al., 2020). Besides providing soluble P to plants, these organisms promote plant growth and development by other activities, such as nitrogen fixation and production of plant phytohormones (Zaidi et al., 2009).

Among the PS mechanisms utilized by PSB, the production of organic acids is well recognized and most common in rhizobacteria. The organic acids excreted by PSB have different modes of action that lead to PS: mineral ions bound to precipitated Pi can be chelated by these organic acids or P can be dissolved by decreasing the pH (Rashid et al., 2004). Among the genera considered as PSB, *Paenibacillus* has shown to be promising for inoculation purposes. *Paenibacillus* members that present PS activity have been isolated from the rhizosphere of various crops (Seldin et al., 1998; Tcherpakov et al., 1999; Beneduzi et al., 2010). In some *Paenibacillus* species such as *Paenibacillus mucilaginosus* (Hu et al., 2006), *Paenibacillus elgii* (Narayan et al., 2010), *Paenibacillus kribbensis* (Marra et al., 2012), *Paenibacillus polymyxa*, *Paenibacillus macerans* (Wang et al., 2012), and *Paenibacillus xylanilyticus* (Pandya et al., 2015), the PS ability has been confirmed. In a comparative genomic analysis, it was suggested that a wide group of *Paenibacillus* can perform PS by producing the organic acid gluconate, as several *Paenibacillus* species (i.a. *P. polymyxa*, *Paenibacillus peoriae*, and *Paenibacillus* sp.) showed high conservation of the genes coding for the enzymes glucose 1-dehydrogenase and gluconate dehydrogenase, which are involved in the synthesis of this compound (Xie et al., 2016).

*Paenibacillus sonchi* genomovar Riograndensis is a rod-shaped, Gram-positive, motile, nitrogen-fixing bacterium (Beneduzi et al., 2010; Sant’Anna et al., 2017). The strain SBR5 was isolated from the rhizosphere of wheat (*Triticum aestivum*) plants cultivated in Rio Grande do Sul, Brazil (Beneduzi et al., 2008). In addition to nitrogen fixation, SBR5 possesses further plant growth-promoting activities, such as the ability to produce indole-3- acetic acid and to excrete a high amount of ammonium (Fernandes et al., 2014; Ambrosini et al., 2018). The complete genome of *P. sonchi* SBR5 (Genbank accession LN831776) was sequenced and fully annotated and consists of one chromosome of 7,893,056 bp, containing 6,705 protein-coding genes, 87 tRNAs, and 27 rRNAs genes (Brito et al., 2015). RNAseq has been used to chart its global transcriptomic landscape (Brito et al., 2017a). Moreover, molecular tools for gene expression (Brito et al., 2017b) and CRISPR interference-based gene repression (Brito et al., 2020) were developed for this organism. Hence, a methodology is in place to study the poorly characterized PS by SBR5. To elucidate the PS process in this bacterium, we performed RNAseq and physiological analyses with two inorganic P sources: soluble and insoluble.

## Materials and Methods

### Strains and Growth Conditions

The PGPR bacterium *P. sonchi* genomovar Riograndensis SBR5 was obtained from the strain collection of the Department of Genetics at Universidade Federal do Rio Grande do Sul, Brazil. SBR5 and *Escherichia coli* DH5α cells were used as expression host and molecular cloning host, respectively. All bacterial strains utilized in the present study are listed in Supplementary Table 1. *E. coli* cells were routinely cultivated in Luria–Bertani medium, composed of 10 g L^–1^ tryptone, 5 g L^–1^ yeast extract, and 10 g L^–1^ NaCl, at 37°C and 120 rpm, and chloramphenicol (20 μg ml^–1^) and ampicillin (100 μg ml^–1^) were added to cultivation broth when necessary. Precultures of SBR5 transformants were routinely cultivated in Caso broth (DSMZ 220), containing 15 g L^–1^ peptone from casein, 5 g L^–1^ peptone from soymeal, and 5 g L^–1^ NaCl, and chloramphenicol (5 μg ml^–1^) and ampicillin (10 μg ml^–1^) were added to medium when necessary. Bacterial cells were grown in Caso broth agar plates, and a single colony was picked and transferred to 500 ml shaking flasks containing 50 ml of the same medium, and the flask was incubated overnight at 30°C and 120 rpm. One milliliter of the bacterial suspension was inoculated into 500 ml shaking flasks containing 50 ml of distinct broth media differing with regard to the P source: (1) Pikovskaya broth prepared as Pikovskaya (1948) with 5 g L^–1^ of Ca_5_(PO_4_)_3_(OH) (hydroxyapatite) as P source (IPi); (2) Pikovskaya broth with 3.2 g L^–1^ NaH_2_PO_4_ as P source (SPi). The pH of the media was adjusted to 7.0 before autoclaving, and the pH neutrality was maintained by the addition of 12 g L^–1^ of 2-[4-(2-hydroxyethyl)piperazin-1-yl]ethanesulfonic acid. The cells grown in each P source were used as inoculum in fresh media of the same condition. For each condition tested, six biological replicates were used: three for total RNA isolation and three for further determination of growth characteristics. Bacterial cells of three replicate cultivations were harvested after 6 h of growth, in the middle of the exponential phase (Supplementary Figure 1). The harvesting procedure was done according to Irla et al. (2016); 20 ml of each sample was collected in 50-ml falcon tubes filled with shaved ice and centrifuged at 4°C for 10 min at 4,000 rpm. The resulting cell pellets were immediately frozen in liquid nitrogen after centrifugation and stored at −80°C until further use. Additionally, SBR5 was cultivated in CGXII minimal medium (Keilhauer et al., 1993) with some modifications. CGXII medium was composed of 20 g L^–1^ ammonium sulfate, 5 g L^–1^ urea, and 12 g L^–1^ 2-[4-(2-hydroxyethyl)piperazin-1-yl]ethanesulfonic acid, with supplementation of a trace salts solution (Keilhauer et al., 1993), 9 g L^–1^ glucose, and 0.1 mg L^–1^ biotin. The phosphate content of the medium varied with gradually increasing concentrations of NaH_2_PO_4_ (0, 13, 26, 39, 52, and 65 mM) to obtain microbial growth kinetics. The precultures were grown in CGXII with either sufficient (70 mM) or depleted (20 mM) Pi. For growth analysis, SBR5 cells were cultivated in IPi, SPi, low phosphate (LPi), and high phosphate (HPi) media. LPi and HPi media were prepared according to Qi et al. (1997), with some modifications. Both media were composed of 4 g L^–1^ ammonium sulfate, 0.18 g L^–1^ sodium citrate, 0.5 g L^–1^ ferric chloride, 0.15 g L^–1^ manganese sulfate, 4.2 g L^–1^ magnesium sulfate, 6 g L^–1^ Trizma, 1.3 mg L^–1^ zinc chloride, and 12.3 mg L^–1^ (LPi) or 0.49 g L^–1^ (HPi) K_2_HPO^4^; both media were supplemented 9 g L^–1^ glucose, 0.5 g L^–1^ casamino acids, and 0.1 mg L^–1^ biotin. Two milliliters of cultivation broth were taken for measurements of optical density at 600 nm (OD_600 *nm*_) and pH (pH indicator sticks, Macherey-Nagel, Düren, Germany), and supernatants and cell pellets (*n* = 3) were collected by centrifugation (10 min at 4,000 rpm). The initial OD_600 *nm*_ of main cultivations was approximately 0.05. During the cultivations, *P. sonchi* cells were routinely shaken at 120 rpm at a temperature of 30°C.

### RNA Isolation and Preparation and Sequencing of Complementary DNA Libraries

To isolate the total RNA from SBR5 cells, bacterial cell pellets previously harvested and stored at −80°C were thawed in ice, and RNA was extracted individually for each cultivation condition. All the procedures regarding RNA isolation and RNA quality control were done according to Brito et al. (2017a). The three replicates of extracted RNA samples of each condition were pooled in equal parts, and the pool of total RNA was subsequently used for the preparation of complementary DNA (cDNA) libraries. For each condition, a whole transcriptome library was prepared. For removal of ribosomal RNA, Ribo-Zero Plus rRNA Depletion Kit (Illumina, San Diego, United States) was used according to manufacturer’s recommendations. The preparation of this library and the cDNA sequencing were carried out according to Mentz et al. (2013).

### Bioinformatics Analysis

The sequence reads were mapped onto the reference genome of *P. sonchi* genomovar Riograndensis SBR5 (Brito et al., 2015). To prepare the reads for mapping, the tool Trimmotatic version 0.33 (Bolger et al., 2014) was used to trim the sequences to a minimal length of 35 bp. Trimmed reads were mapped to the reference genome of SBR5 through the software for short read alignment Bowtie (Langmead, 2010). The visualization of mapped reads and the differential gene expression analysis were performed using the software ReadXplorer (Hilker et al., 2014), in which the statistical method DEseq was used to analyze the resultant RNAseq data (Anders and Huber, 2010). The cutoff values for designating a gene as differentially expressed included a change in expression level (base mean) higher than 30 and an adjusted *P*-value equal to or less than 0.05. When differentially expressed genes coding for proteins of unknown function were detected, the gene sequences were submitted to BLASTx analysis to identify the protein family conservations (Altschup et al., 1990).

### Real-Time Quantitative Reverse Transcription-Polymerase Chain Reaction

To validate the data obtained by RNAseq analysis, real-time quantitative reverse transcription-polymerase chain reaction (qRT-PCR) was performed utilizing a LightCycler system (Roche Diagnostics, Penzberg, Germany). The RNA samples utilized to compose the RNA pools for cDNA library preparation in this study were also utilized as templates for qRT-PCR. Differentially expressed genes selected for this assay and the characteristics of the primers utilized are listed in Supplementary Table 2. Furthermore, we selected the genes *glpQ*, *phoH*, *phoU*, *pstB3*, and *pstS*, which are differentially expressed in the condition of Pi starvation in bacteria (Baek and Lee, 2007; Myers et al., 2016; Devine, 2018), to be targeted in the qRT-PCR analysis. Therefore, to obtain messenger RNA from SBR5 in Pi starvation conditions, SBR5 cells were cultivated in HPi medium until they reached exponential phase and subsequently transferred to LPi medium or HPi control medium for RNA isolation at the times 15, 60 min, 6, and 24 h. For analysis of the isolated RNA, qRT-PCR was performed utilizing a CFX Connect Real-Time PCR Detection System (BioRad Laboratories, Hercules, United States). Therefore, each sample concentration was adjusted to 50 ng RNA μl^–1^, and 1 μl thereof was pipetted into a reaction mixture of the SensiFAST SYBR No-ROX Kit (Bioline, Luckenwalde, Germany), following manufacturer’s instructions. To evaluate relative gene expression, 16S rRNA was used as reference control (Sperb et al., 2016). The melting-curve data-based quantification cycle (Cq) values were used for further calculation. For the calculation of relative gene expression, the following equation was used: ΔΔCq = 2^–(Cq^*^*IpI*^* ^–^^*Cq*^*^16S)^* / 2^–(CqSPi^^–^^*Cq16S)*^ (Livak and Schmittgen, 2001).

### Molecular Cloning, Plasmid DNA Construction, and Preparation of Recombinant Strains

Molecular cloning was performed as described previously (Sambrook, 2001). Chemically competent cells of *E. coli* DH5α were prepared for cloning (Hanahan and Harbor, 1983). All plasmids obtained in this study and all oligonucleotide sequences utilized in PCR are listed in Supplementary Table 1. Genomic DNA of *P. sonchi* SBR5 was isolated using NucleoSpin Microbial DNA kit (Machery-Nagel, Düren, Germany). The NucleoSpin Gel and PCR Clean-up kit (Machery-Nagel) was used for PCR cleanup, and plasmids were isolated using the GeneJET Plasmid Miniprep Kit (Thermo Fisher Scientific, Waltham, United States). Plasmid backbones were digested using restriction enzyme BamHI (Thermo Fisher Scientific). Double-stranded DNA inserts were amplified using Phusion DNA polymerase (New England Biolabs, Ipswich, England) and the overlapping regions joined by Gibson assembly (Gibson, 2014). For colony PCR, Taq polymerase (New England Biolabs) was used according to the manufacturer’s recommendations. The sequence correctness of resultant plasmids was checked by sequencing in the Center for Biotechnology in Bielefeld, Germany. Plasmid DNA was used to transform *P. sonchi* SBR5 cells as in Brito et al. (2017b).

### Quantification of Free Inorganic Orthophosphates in Cell Pellets and Supernatants

PS efficiency of SBR5 cultivated in the different conditions (IPi, SPi, LPi, and HPi) was determined through the quantification of orthophosphates present in the supernatants or cell lysates. The cells were lysed by ultrasonication for 6 min at on and off cycles of 0.5 s and amplitude 55% (Ultraschalldesintegrator Sonoplus GM 200, Sonotrode M72, Bandelin electronic GmbH & Co KG, Berlin, Germany) and centrifuged for 30 min at 4°C and 13,200 rpm. This assay was performed through the molybdenum-blue method, as described by Murphy and Riley (1958).

### Metabolite Analyses

For quantification of glucose and organic acids in the supernatants and cell lysates of SBR5, a high-performance liquid chromatography system (HPLC, 1200 series, Agilent Technologies Deutschland GmbH, Böblingen, Germany) was used as in Zahoor et al. (2014). Quantification was done by calibration with external standards. By this method, gluconate, oxoglutarate, acetate, citrate, succinate, oxalate, and malate of cell supernatants at 20 h, at the end of RNAseq cultivation (Supplementary Figure 1), were determined. Moreover, oxoglutarate, trehalose, and glycine betaine were also measured in supernatants and cell lysates, along with the growth of SBR5 in IPi and SPi. Furthermore, 2-oxoglutarate dehydrogenase (2-OGDH) activity of SBR5 crude extracts in IPi or SPi was measured according to Nguyen et al. (2015). To obtain the concentration of amino acids present in cell supernatants of SBR5 at the end of its growth in RNAseq cultivation (Supplementary Figure 1), HPLC samples were derivatized with *ortho*-phthaldialdehyde and separated on a system consisting of a pre-column (LiChrospher 100 RP8 EC-5μ, 40 × 4.6 mm, CS-Chromatographie Service GmbH, Langerwehe, Germany) and the main column (LiChrospher 100 RP8 EC-5μ, 125 × 4.6 mm, CS-Chromatographie Service GmbH, Langerwehe, Germany) and detected by fluorescence (FLD G1321A, 1200 series, Agilent Technologies) (Jorge et al., 2017b). Asparagine was used as an internal standard, and external standards of glutamate, valine, arginine, and ornithine were prepared for calibration. For proline measurement, samples were derivatized with 9-fluorenylmethyl chloroformate as in Jensen et al. (2015). Furthermore, trehalose, glycine betaine, oxoglutarate, proline, and glutamate were measured in supernatants and cell lysates of SBR5 along with its growth in SPi or IPi.

### Determination of Osmolality Levels

Osmolality measurements of IPi and SPi samples (supernatant and lysates from cell pellets) were done by freezing point depression using an Osmomat auto (Gonotec, Berlin, Germany) according to the manufacturer’s instructions. Supernatant samples and cell lysates were directly used for the measurement. The total osmolality of each sample was determined by comparative measurements of the freezing points of pure water and the test solutions using 50 μl of the sample.

### Flow Cytometry

Recombinant *P. sonchi* SBR5 cells were cultivated in SPi or IPi supplemented with chloramphenicol or ampicillin. Cells were harvested in the mid-exponential phase of growth and washed three times in NaCl 0.9%. Next, the OD_600 *nm*_ of the samples was normalized to 0.5. To quantify the fluorescence intensities in the cell suspensions, they were submitted to flow cytometry (Beckman Coulter, Brea, United States) and the data analyzed in the Beckman Coulter Kaluza Flow Analysis Software. The settings for the emission signal and filters within the flow cytometer for detection of GfpUV and Crimson fluorescence were 550/525 bandpass FL9 filter and 710/660 bandpass FL6 filter, respectively.

### Statistical Analysis

Student’s *t*-test was used to assess statistical differences in mean orthophosphate concentrations over time in the supernatants of SBR5 cultivated in IPi condition. Results were considered statistically significant when *p*-values were <0.01 or <0.001.

## Results

### Growth and Gene Expression of *P. sonchi* SBR5 With Different Pi Concentrations

Growth of *P. sonchi* SBR5 in CGXII supplemented with increasing Pi concentrations (0-, 13-, 26-, 39-, 52-, and 65-mM NaH_2_PO_4_) was compared, with precultures grown with sufficient (70 mM) or depleted (20 mM) Pi. Only sub-millimolar Pi concentrations reduced growth when precultures were grown with sufficient Pi (data not shown), which indicated that an inner cell P storage, such as polyphosphate granule (Racki et al., 2017), may exist. Therefore, precultures were grown in depleted Pi. A Monod constant for NaH_2_PO_4_ of approximately 10 mM was determined, as this concentration supported a half-maximal growth rate (Supplementary Figure 2). The final biomass (ΔOD) of SBR5 cells was negatively affected by Pi depletion when the supplied NaH_2_PO_4_ concentration was inferior to 26 mM (Supplementary Figure 2).

**FIGURE 1:**
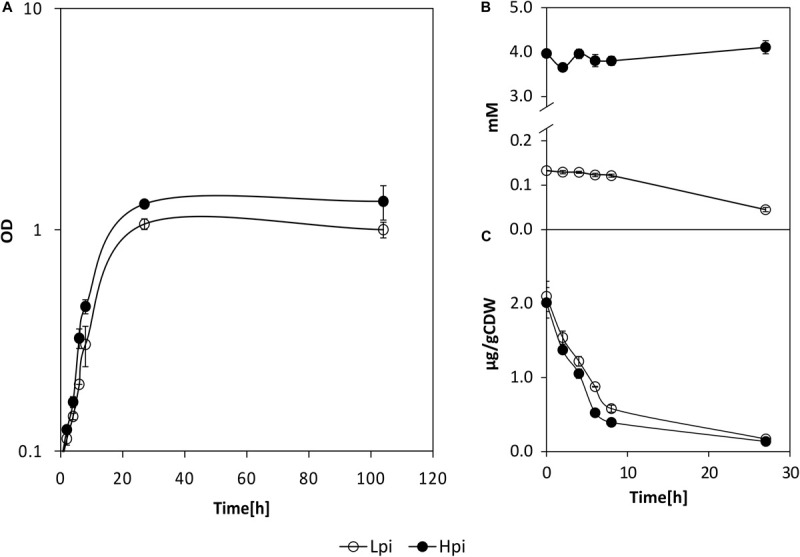
Growth **(A)** and orthophosphate concentrations in the supernatants **(B)** and cell lysates **(C)** of *P. sonchi* SBR5 cultivated in high phosphate medium (HPi, closed circles) or low phosphate medium (LPi, open circles). CDW, cell dry weight. Data given as means and standard deviations of biological triplicates.

To analyze a Pi down shock, precultures grown in HPi were transferred to HPi or LPi media, containing 5 or 0.13 mM of Pi, respectively. LPi condition is expected to be similar to IPi condition with respect to the soluble Pi concentration in the culture broth. During growth in LPi and HPi conditions, pH values of supernatant remained neutral (data not shown), and growth in LPi condition was only slightly lower than in HPi (Figure 1A). The Pi concentration in the supernatants decreased 30% during growth in LPi condition (Figure 1B), whereas the intracellular Pi concentration dropped drastically during growth under both conditions (Figure 1C).

**FIGURE 2:**
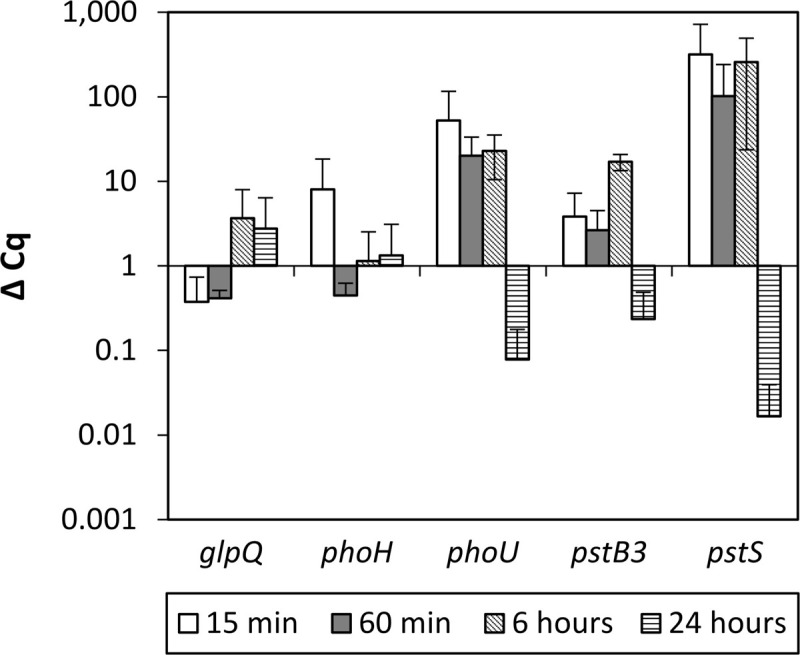
qRT-PCR analysis of *P. sonchi* SBR5 in low phosphate shock, grown in high phosphate medium (HPi) and transferred to low phosphate medium (LPi). ΔCq values for the indicated genes (*glpQ*, *phoH*, *phoU*, *pstB3*, and *pstS*), and the indicated RNA harvesting times after transferring are given as means and standard deviations of biological triplicates.

To verify if Pi inducible genes known from other bacteria (*glpQ*, *phoH*, *phoU*, *pstB3*, and *pstS*) (Baek and Lee, 2007; Myers et al., 2016; Devine, 2018) show increased RNA levels in SBR5 upon transfer from HPi to LPi culture broth, *P. sonchi* SBR5 cells cultivated in HPi medium were transferred to HPi or LPi, and RNA samples were taken after 15, 60 min, 6, or 24 h for qRT-PCR analysis. All the targeted genes showed increased RNA levels upon transfer to the LPi medium (Figure 2). The genes *pstB3* and *pstS*, coding for components of the high-affinity Pi transport system, and the gene *phoU*, encoding a phosphate-specific transport system accessory protein, were highly expressed 15, 60 min, and 6 h after the transfer to LPi condition but downregulated at 24 h. The gene *phoH*, coding for a Pi starvation protein, was expressed only at 15 min after the transfer to LPi condition, whereas the glycerophosphodiester phosphodiesterase gene *qlpQ* showed increased ΔCq levels at 6 and 24 h after the transfer to LPi condition. Pi induction of *pstS* was strongest (approximately hundredfold after the transfer from HPi to LPi condition; Figure 2), indicating *pstS* as a good indicator of Pi starvation.

### Growth of *P. sonchi* SBR5 in Soluble (SPi) and Insoluble (IPi) Containing P Media

**FIGURE 3:**
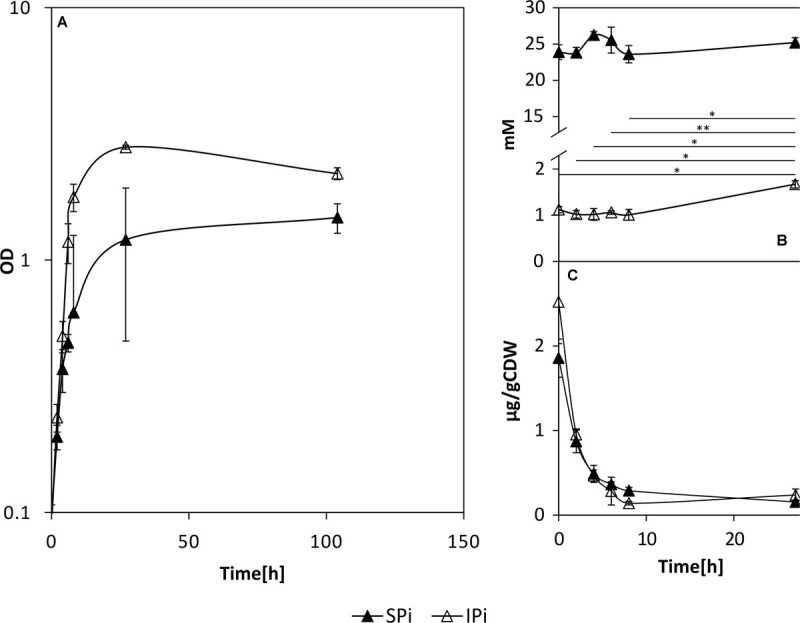
Growth **(A)** and orthophosphate concentrations in the supernatants **(B)** and cell lysates **(C)** of *P. sonchi* SBR5 cultivated with IPi or SPi. Pikovskaya broth (Pikovskaya, 1948) was used for growth with IPi, and Pikovskaya broth with phosphate source replaced by NaH_2_PO_4_ for growth with SPi. Data are given as means and standard deviations of biological triplicates. Student’s paired *t*-test was performed to examine the significance level and error probability (*p < 0.01; **p < 0.001). CDW, cell dry weight.

Growth of *P. sonchi* SBR5 in either 5 g L^–1^ hydroxyapatite (IPi) or 30 mM NaH_2_PO_4_ (SPi) was compared with precultures grown with depleted Pi. The maximum growth rate under both conditions was comparable (0.18 h^–1^, data not shown), but SBR5 grew to a higher biomass concentration (OD_600 nm_) under the IPi condition (Figure 3A). During RNAseq cultivation, the medium pH dropped, which was more prominent in IPi medium (pH dropped from 7 ± 0 to 4 ± 0) in comparison with SPi medium (from 7 ± 0 to 6 ± 0) (Supplementary Figure 1). SBR5 cultures cultivated in the IPi and SPi conditions represented in Figure 3 presented the same effect in pH (data not shown).

The P source in IPi was 5 g L^–1^ hydroxyapatite, which was not promptly available for the cells in the first hours of growth (Figure 3B). In the first hours of growth, the supernatants of cells cultivated in IPi contained approximately 1 mM of orthophosphates. This is approximately 20 times less than the concentration of Pi present in supernatants of cells cultivated in SPi, in which the Pi concentration was kept relatively constant (∼22 mM) along with the growth of SBR5. Nevertheless, the concentration of orthophosphates in the supernatants increased to approximately 2 mM at 24 h. The difference between the initial and final concentration of Pi in supernatants of IPi condition shows an increase of approximately 1 mM of orthophosphates in IPi medium during the growth of *P. sonchi* SBR5 (Figure 3B). Cell lysate orthophosphate concentrations under SPi and IPi conditions were drastically depleted in the first hours of growth (Figure 3C).

### Differential Gene Expression Analysis and Validation of Expression Pattern by Real-Time Quantitative Reverse Transcription-Polymerase Chain Reaction

Gene expression analysis regarding the P metabolism and PS in bacteria has previously been performed through microarray (Bianco and Defez, 2010) and RNAseq technologies (Zeng et al., 2017). Here, we carried out RNASeq analysis to determine differential gene expression of *P. sonchi* SBR5 cultivated with a soluble P source (NaH_2_PO_4_, SPi medium) and an insoluble P source (hydroxyapatite, IPi medium). Sequencing of cDNA libraries generated from RNA obtained from those cultures resulted in 2,729,614 reads for the SPi sample and 2,773,600 reads for the IPi sample. Of the resultant reads, 2,596,992 and 2,611,860 reads of SPi and IPi libraries, respectively, were mapped onto the genome of SBR5 (Supplementary Table 3). Our DESeq analysis revealed higher expression of 68 genes in IPi (Table 1) and reduced expression of 100 genes in IPi as compared with SPi condition (Table 2), as represented in Supplementary Figure 3.

**TABLE 1:** List of *P. sonchi* SBR5 genes showing increased RNA levels during cultivation with IPi in comparison to SPi.

**Feature**	**Product**	**log_2_ Fold Change**	***P*-value**
P.riograndensis_final_1089	Conserved hypothetical membrane protein	>6	0.01
P.riograndensis_final_1858	Integral membrane sensor signal transduction histidine kinase	>6	0.02
P.riograndensis_final_233	Conserved hypothetical protein	>6	0.03
P.riograndensis_final_297	Putative drug resistance ABC-2 type transporter, permease protein	>6	0.03
P.riograndensis_final_6163	Conserved hypothetical protein	5.71	0.00
*opuAA*	Glycine betaine transport ATP-binding protein OpuAA	5.00	0.00
P.riograndensis_final_2300	Transcriptional regulator TenI	4.99	0.00
P.riograndensis_final_3056	Conserved hypothetical secreted protein	4.81	0.00
P.riograndensis_final_6139	Hypothetical protein	4.72	0.00
P.riograndensis_final_5641	Glycine betaine transport system permease protein opuAB	4.53	0.00
P.riograndensis_final_5637	Hypothetical protein	4.52	0.00
P.riograndensis_final_6503	Conserved hypothetical protein	4.04	0.03
P.riograndensis_final_6164	Flagellin	4.00	0.00
P.riograndensis_final_5029	Hypothetical membrane protein	3.97	0.01
P.riograndensis_final_6162	Flagellar capping protein	3.95	0.00
P.riograndensis_final_5640	ABC transporter, substrate-binding protein, QAT family	3.91	0.00
P.riograndensis_final_1047	NUDIX hydrolase	3.61	0.05
P.riograndensis_final_4130	Pyrroline-5-carboxylate reductase	3.47	0.04
P.riograndensis_final_6151	–	3.42	0.01
P.riograndensis_final_3685	Methyl-accepting chemotaxis protein	3.42	0.04
*motA*	Motility protein A	3.33	0.01
P.riograndensis_final_6184	Predicted protein	3.16	0.01
P.riograndensis_final_6178	Conserved hypothetical membrane protein	3.14	0.01
P.riograndensis_final_2303	Thiazole biosynthesis protein ThiH	3.13	0.01
P.riograndensis_final_2717	Integral membrane sensor signal transduction histidine kinase	3.12	0.01
*mcp4*	Methyl-accepting chemotaxis protein	3.09	0.03
P.riograndensis_final_2867	Methyl-accepting chemotaxis sensory transducer	3.07	0.04
*rplU*	50S ribosomal protein L21	3.05	0.01
*fliH*	Flagellar assembly protein FliH	3.03	0.01
P.riograndensis_final_6160	Conserved hypothetical protein	3.03	0.02
P.riograndensis_final_518	Conserved hypothetical membrane protein	2.98	0.02
P.riograndensis_final_4041	Signal peptidase I (SPase I) (Leader peptidase I)	2.95	0.01
P.riograndensis_final_1993	Hypothetical protein	2.91	0.03
*blt*	Multidrug resistance protein 2	2.88	0.05
*thiG*	Thiazole synthase	2.85	0.02
P.riograndensis_final_3782	Hypothetical protein	2.85	0.03
P.riograndensis_final_6161	Flagellar protein FliS	2.85	0.02
P.riograndensis_final_6026	Ribosomal protein S10	2.83	0.01
P.riograndensis_final_2999	Hypothetical protein	2.78	0.02
P.riograndensis_final_3001	ABC transporter ATP-binding protein	2.76	0.04
P.riograndensis_final_6158	Hypothetical protein	2.74	0.02
P.riograndensis_final_3212	Conserved hypothetical protein	2.72	0.04
P.riograndensis_final_5299	Conserved hypothetical protein	2.72	0.02
*thiE*	Thiamine-phosphate synthase	2.71	0.04
P.riograndensis_final_4022	Flagellar basal-body rod protein flgC	2.65	0.02
*thiD*	Hydroxymethylpyrimidine/phosphomethylpyrimidinekinase	2.64	0.03
*rkpK*	UDP-glucose 6-dehydrogenase	2.62	0.03
*queE*	7-Carboxy-7-deazaguanine synthase	2.61	0.03
*fliI*	Flagellum-specific ATP synthase	2.61	0.03
P.riograndensis_final_6154	–	2.57	0.03
*motB*	Motility protein B	2.54	0.04
P.riograndensis_final_5386	Hypothetical protein	2.53	0.03
P.riograndensis_final_1949	Methyl-accepting chemotaxis sensory transducer	2.53	0.05
*fliG*	Flagellar motor switch protein FliG	2.52	0.03
P.riograndensis_final_2718	LuxR family two component transcriptional regulator	2.52	0.04
P.riograndensis_final_2301	–	2.50	0.04
P.riograndensis_final_5926	Amino acid ABC transporter permease	2.48	0.04
*tetB*	Tetracycline resistance protein	2.45	0.04
P.riograndensis_final_4023	Conserved hypothetical protein	2.41	0.04
P.riograndensis_final_5390	NAD-dependent epimerase/dehydratase	2.41	0.05
*flhB*	Flagellar biosynthetic protein FlhB	2.39	0.04
P.riograndensis_final_4015	Hypothetical protein	2.37	0.04
P.riograndensis_final_4021	Hypothetical protein	2.36	0.04
P.riograndensis_final_5927	Capsule synthesis protein, CapA	2.34	0.04
P.riograndensis_final_4020	Flagellar M-ring protein FliF	2.28	0.04
P.riograndensis_final_4042	Ribosomal protein L19	2.22	0.05
*thiC*	Phosphomethylpyrimidine synthase	2.21	0.05
P.riograndensis_final_3098	Hypothetical protein	2.19	0.05

**TABLE 2:** List of *P. sonchi* SBR5 genes showing reduced RNA levels during cultivation with IPi in comparison to SPi.

**Feature**	**Product**	**log_2_ Fold Change**	***P*-value**
P.riograndensis_final_1900	Hypothetical protein	−5.22	0.00
P.riograndensis_final_1813	ABC transporter	−5.15	0.00
P.riograndensis_final_345	Conserved hypothetical protein	−4.92	0.00
P.riograndensis_final_4843	Hypothetical protein	−4.78	0.00
P.riograndensis_final_4161	Conserved hypothetical secreted protein	−4.65	0.00
P.riograndensis_final_5412	Phosphate binding protein	−4.63	0.00
*yusV*	Probable siderophore transport system ATP- binding protein YusV	−4.61	0.00
P.riograndensis_final_1897	Transport system permease	−4.52	0.00
P.riograndensis_final_1812	ABC-type multidrug transport system	−4.47	0.00
P.riograndensis_final_2959	Hypothetical protein	−4.46	0.00
*ywbG*	Uncharacterized protein YwbG	−4.35	0.00
P.riograndensis_final_5808	Transcription cofactor, metal binding pirin domain protein	−4.12	0.00
P.riograndensis_final_5825	Hypothetical membrane protein	−4.10	0.00
*yugH*	Putative aminotransferase YugH	−4.07	0.00
P.riograndensis_final_5413	Conserved hypothetical protein	−4.07	0.01
P.riograndensis_final_344	Binding-protein-dependent transport systems inner membrane component	−4.04	0.05
P.riograndensis_final_1811	S-layer domain-containing protein	−4.02	0.00
P.riograndensis_final_3815	Hypothetical protein	−4.00	0.00
P.riograndensis_final_603	Conserved hypothetical protein	−3.99	0.00
P.riograndensis_final_1123	Conserved hypothetical protein	−3.94	0.00
*phaA*	Acetyl-CoA acetyltransferase	−3.82	0.05
P.riograndensis_final_602	Hypothetical protein	−3.81	0.00
P.riograndensis_final_4326	Conserved hypothetical protein	−3.70	0.00
P.riograndensis_final_1896	Periplasmic-binding protein	−3.63	0.00
P.riograndensis_final_5813	Conserved hypothetical membrane protein	−3.63	0.00
P.riograndensis_final_2763	Hypothetical protein	−3.63	0.01
P.riograndensis_final_3666	PAS domain S-box protein	−3.62	0.00
P.riograndensis_final_5814	Conserved hypothetical membrane protein	−3.53	0.00
*treA*	Trehalose-6-phosphate hydrolase	−3.45	0.01
P.riograndensis_final_346	Conserved hypothetical membrane protein	−3.44	0.01
P.riograndensis_final_1182	YhgE/Pip N-terminal domain protein	−3.41	0.00
P.riograndensis_final_3382	Hypothetical protein	−3.31	0.02
P.riograndensis_final_3123	General stress protein 16O	−3.29	0.01
P.riograndensis_final_2934	Hypothetical protein	−3.29	0.02
P.riograndensis_final_1712	Glutamine–scyllo-inositol transaminase	−3.28	0.01
P.riograndensis_final_1769	ABC transporter, permease protein	−3.28	0.01
P.riograndensis_final_1898	Transport system permease	−3.28	0.01
*fabG*	3-Ketoacyl-ACP reductase	−3.28	0.01
P.riograndensis_final_2070	Trypsin	−3.25	0.01
P.riograndensis_final_1553	Hypothetical protein	−3.17	0.01
*amyD*	Probable starch degradation products transport system permease protein AmyD	−3.14	0.02
P.riograndensis_final_1679	Non-ribosomal peptide synthase/amino acid adenylation enzyme	−3.11	0.01
P.riograndensis_final_635	Conserved hypothetical secreted protein	−3.10	0.01
P.riograndensis_final_2339	Hypothetical protein	−3.09	0.01
P.riograndensis_final_1768	ABC transporter substrate-binding protein	−3.08	0.01
*odhB*	Dihydrolipoyllysine-residue succinyltransferase component of 2-oxoglutarate dehydrogenase complex	−3.05	0.01
P.riograndensis_final_3118	Hypothetical protein	−3.05	0.01
P.riograndensis_final_6290	Conserved hypothetical membrane protein	−3.04	0.01
P.riograndensis_final_1238	Hypothetical protein	−3.04	0.01
*yunF*	UPF0759 protein YunF	−3.04	0.01
P.riograndensis_final_3303	–	−3.03	0.03
*odhA*	2-Oxoglutarate dehydrogenase E1 component	−3.00	0.01
*pstS*	Phosphate-binding protein PstS	−2.96	0.02
P.riograndensis_final_1377	Hypothetical membrane protein	−2.93	0.02
P.riograndensis_final_2342	Conserved hypothetical protein	−2.86	0.01
P.riograndensis_final_2340	Hypothetical protein	−2.82	0.01
P.riograndensis_final_2126	Ferritin Dps family protein	−2.80	0.01
P.riograndensis_final_1699	Putative polyketide synthase component, beta- ketoacyl synthase family protein	−2.79	0.01
P.riograndensis_final_1680	Cyclic peptide transporter	−2.75	0.02
P.riograndensis_final_1682	Possible methoxymalonyl-ACP biosynthesis protein	−2.73	0.02
P.riograndensis_final_483	Hypothetical protein	−2.72	0.02
P.riograndensis_final_613	Conserved hypothetical protein	−2.71	0.03
*yhxC*	Uncharacterized oxidoreductase YhxC	−2.71	0.02
*ytcD*	Hypothetical protein	−2.69	0.02
*hbd*	3-Hydroxybutyryl-CoA dehydrogenase	−2.68	0.02
P.riograndensis_final_1717	Putative transcription antitermination protein	−2.67	0.02
*treP*	Alpha,alpha-trehalose phosphorylase	−2.67	0.03
P.riograndensis_final_1711	Conserved hypothetical protein	−2.63	0.03
P.riograndensis_final_1501	Hypothetical protein	−2.63	0.02
P.riograndensis_final_1710	Conserved hypothetical protein	−2.63	0.03
*sodA*	Superoxide dismutase [Mn]	−2.61	0.02
P.riograndensis_final_2002	Conserved hypothetical membrane protein	−2.59	0.03
P.riograndensis_final_3384	Hypothetical protein	−2.59	0.03
P.riograndensis_final_4181	Hypothetical protein	−2.59	0.04
P.riograndensis_final_2341	Hypothetical protein	−2.58	0.02
*glgC*	Glucose-1-phosphate adenylyltransferase	−2.57	0.02
P.riograndensis_final_681	Conserved hypothetical protein	−2.56	0.03
*sufB*	FeS cluster assembly protein SufB	−2.56	0.03
P.riograndensis_final_466	Hypothetical protein	−2.53	0.02
P.riograndensis_final_3694	Alpha/beta hydrolase	−2.53	0.03
P.riograndensis_final_3385	AstB/chuR/nirj-like protein	−2.53	0.03
P.riograndensis_final_3383	Hypothetical protein	−2.51	0.03
P.riograndensis_final_5414	Conserved hypothetical secreted protein	−2.50	0.03
P.riograndensis_final_3113	Short-chain dehydrogenase/reductase SDR	−2.49	0.03
P.riograndensis_final_6333	Glucan 1,4-alpha-maltohexaosidase	−2.47	0.03
*fhuC*	Iron(3+)-hydroxamate import ATP-binding protein FhuC	−2.46	0.03
*sigF*	RNA polymerase sigma-F factor	−2.44	0.04
P.riograndensis_final_1700	Haloacid dehalogenase superfamily protein	−2.41	0.04
P.riograndensis_final_2791	−	−2.39	0.03
P.riograndensis_final_3672	Hypothetical protein	−2.39	0.04
*glgA*	Glycogen synthase	−2.33	0.04
*ampS*	Aminopeptidase AmpS	−2.32	0.04
P.riograndensis_final_1701	−	−2.32	0.04
P.riograndensis_final_5491	Dyp-type peroxidase family protein	−2.30	0.04
P.riograndensis_final_1708	Conserved hypothetical protein	−2.28	0.04
P.riograndensis_final_5492	High-affinity Fe2+/Pb2+ permease	−2.25	0.04
P.riograndensis_final_5805	Alpha/beta hydrolase fold protein	−2.24	0.04
*yhfW*	Putative Rieske 2Fe–2S iron-sulfur protein YhfW	−2.24	0.05
*ylaK*	Uncharacterized protein YlaK	−2.21	0.05
P.riograndensis_final_5826	Conserved hypothetical secreted protein	−2.20	0.05

The results obtained in RNAseq were confirmed by the analysis of gene expression patterns by qRT-PCR for 12 genes selected from the identified differentially expressed genes. The expression patterns of the 12 candidate genes detected by qRT-PCR were in accordance with the gene expression patterns obtained in the RNAseq analysis (Supplementary Figure 4), demonstrating the reliability of our RNAseq analysis.

Differential expression of genes coding for the two *pho* regulon components PhoP-PhoR was not observed in this work; however, the gene for the phosphate-binding protein PstS was downregulated (Table 2). Interestingly, the expression of two genes related to central carbon metabolism was reduced under IPi condition: the *odhAB* operon that codes for two subunits of the 2-OGDH (Table 2). Similarly, carbohydrate storage genes, i.e., *treA* coding for trehalose-6-phosphate hydrolase, *treP* coding for trehalose phospholyase, and *glgA* and *glgC*, coding for glucose-1-phosphate adenylyltransferase and glycogen synthase, respectively, showed reduced expression under IPi condition (Table 2). By contrast, most SBR5 genes involved in thiamine biosynthesis were upregulated in IPi condition: *thiH*, *thiG*, *thiC*, *thiD*, and *thiE* (Table 1). Moreover, the increased expression of the gene coding for capsule synthesis protein CapA (Table 1) may indicate a stress response (Candela and Fouet, 2006). The expression of an operon comprising genes coding for flagellar protein FliS (P.riograndensis_final_6161), flagellar capping protein (P.riograndensis_final_6162), a hypothetical protein similar to FlaG protein (P.riograndensis_final_6163), and a flagellin protein (P.riograndensis_final_6164) was upregulated under IPi condition (Table 1). In addition, eight genes related to flagellation and motility were also upregulated: *motAB* genes coding for motility proteins A and B, flagellar basal body rod protein gene *flgC* (P.riograndensis_final_4022), flagellar biosynthetic protein gene *flhB*, and genes *fliFGHI* coding for flagellar M-ring protein (P.riograndensis_final_4020), flagellar motor switch protein, flagellar assembly protein FliH, and flagellum-specific ATP synthase (Table 1). Furthermore, expression of an operon that includes two genes related to iron and siderophore metabolism (*yusV*-P.riograndensis_final_1900) was downregulated during cultivation in IPi medium (Table 2). Finally, one gene encoding for pyrroline-5-carboxylase reductase (*proC*) and an operon comprising genes related to the transport of glycine betaine (*opuAA* and *opuAB*-P.riograndensis_final_5641) were upregulated in SBR5 grown in IPi condition (Table 1).

### Promoter Activity Assays Using Transcriptional Fusions to Promoterless Genes for Fluorescent Proteins and Flow Cytometry

**FIGURE 4:**
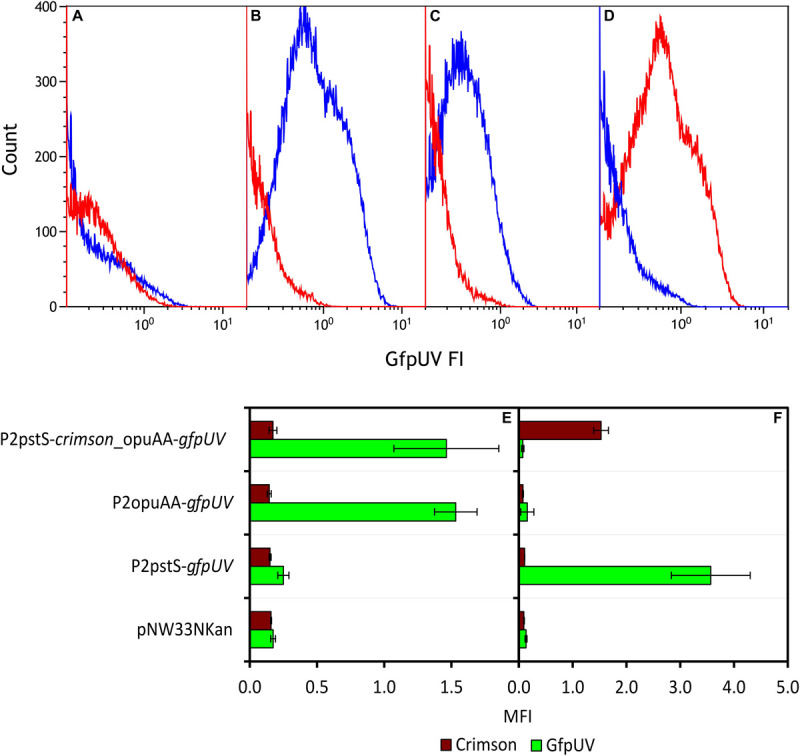
Expression analysis of various promoters in *P. sonchi* SBR5 grown in IPi or SPi. **(A–D)** Histograms show flow cytometry results of GfpUV fluorescence intensity (GfpUV FI) of SBR5 carrying different vectors (**A:** pNW33N; **B:** P2pstS-*gfpUV*; **C:** P2odhA-*gfpUV*; **D:** P2opuAA-*gfpUV*) cultivated in IPi (red) and SPi (blue). **(E,F)** Crimson and GfpUV mean fluorescence intensities (MFI) of SBR5 carrying vectors pNW33N, P2pstS-*gfpUV*, P2odhA-*gfpUV*, P2opuAA-*gfpUV*, and P2pstS-*crimson*_opuAA-*gfpUV* cultivated in IPi **(E)** or SPi **(F)**. MFIs are given as means and standard deviations of triplicate cultivations measured by flow cytometry of 20,000 cells.

The promoterless *gfpUV* reporter gene was fused with promoter sequences of genes upregulated (*opuAA*) or downregulated (*pstS* and *odhA*) under IPi condition. *P. sonchi* strains P2opuAA-*gfpUV*, P2pstS-*gfpUV*, P2odhA-*gfpUV*, and empty vector control pNW33Nkan were cultivated in SPi or IPi media and analyzed by flow cytometry means. SBR5(pNW33Nkan) strain presented GfpUV background signal in both cultivation conditions (Figure 4A). The GfpUV fluorescence intensities of SBR5(P2pstS-*gfpUV*) and SBR5(P2odhA-*gfpUV*) were lower in IPi as compared with SPi condition (Figures 4B,C). In contrast, the GfpUV fluorescence intensity of SBR5(P2opuAA-*gfpUV*) was higher in IPi as compared with SPi condition (Figure 4D). For further corroboration, a strain was constructed to simultaneously monitor the activity of *opuAA* gene promoter (fused to promoterless red-fluorescence reporter gene *crimson*) and *pstS* promoter (fused to promoterless *gfpUV*). Flow cytometry revealed *opuAA* promoter activity in cells cultivated in IPi, whereas *pstS* promoter activity was not detected (Figure 4E). By contrast, when cultivated in SPi, promoter activity was detected for *pstS* but not for *opuAA* (Figure 4F). Thus, reporter gene expression analysis confirmed that *opuAA* is induced under IPi condition, whereas *pstS* and *odhA* gene expression is lower under IPi condition compared with SPi condition.

### High-Performance Liquid Chromatography Analyses: Carbon Metabolism and Osmoprotection

**FIGURE 5:**
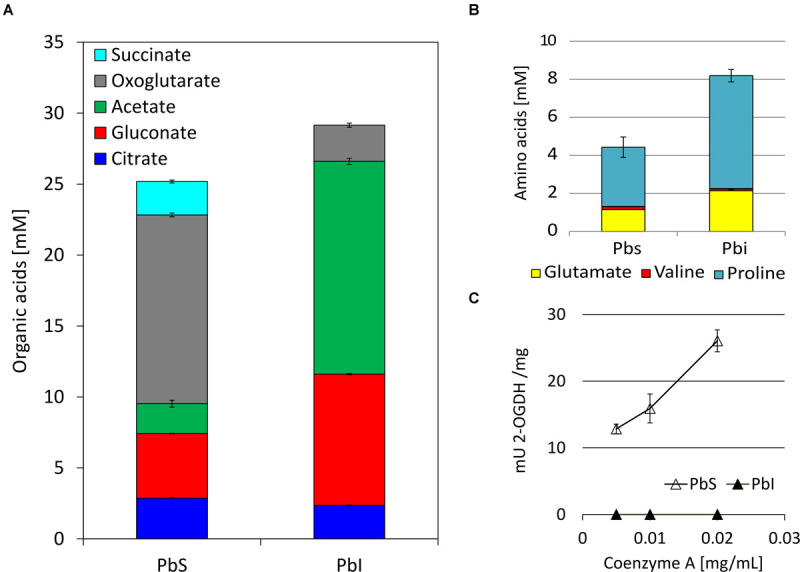
Accumulation of organic acids **(A)** and amino acids **(B)** in the culture medium and 2-oxoglutarate dehydrogenase (2-OGDH)-specific activities (mU 2-OGDH/mg) in crude extracts **(C)** of *P. sonchi* SBR5 cultivated with IPi medium in comparison with SPi. Concentrations of organic acids and amino acids were determined by HPLC analysis of the supernatants collected after 20 h of growth. Arginine and ornithine could not be detected. Activity of 2-OGDH was measured with different concentrations of CoA (milligrams per milliliter). Data are given as means and standard deviations of biological triplicates.

Because the excretion of organic acids by PSB is considered a crucial factor in PS (Rashid et al., 2004), the production of organic acids by SBR5 was monitored under IPi and SPi conditions. A different composition of organic acids was detected in supernatants of SBR5 when this organism was cultivated in IPi or SPi conditions (Figure 5A). The organic acids succinate, oxoglutarate, and citrate, which are components of the tricarboxylic acid (TCA) cycle, were present in the supernatants of SBR5 in SPi condition (Figure 5A). However, in the IPi condition, the concentration of succinate and oxoglutarate decreased 100 and 81%, respectively, in comparison with SPi condition, whereas the citrate concentration was similar to that in the SPi condition. Besides, the amount of acetate and gluconate was 85 and 48% higher, respectively, when SBR5 was cultivated in IPi as compared with SPi (Figure 5A). Arginine and ornithine could not be detected (Figure 5B). Moreover, the 2-OGDH-encoding genes *odhAB* were downregulated when SBR5 was cultivated in IPi condition (Table 2). This result was confirmed by enzymatic assay of 2-OGDH in crude extracts of SBR5, in which 2-OGDH was inactive in crude extracts of cells grown in IPi, whereas activity was observed in SPi condition (Figure 5C).

**FIGURE 6:**
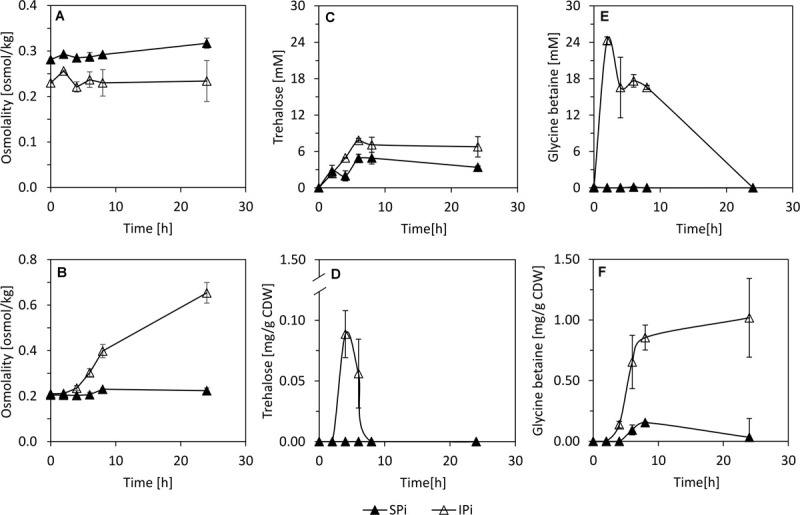
Supernatant **(A,C,E)** and intracellular **(B,D,F)** osmolarity, trehalose, and glycine betaine concentrations during growth of *P. sonchi* SBR5 with IPi and Spi. Pikovskaya broth (Pikovskaya, 1948) was used for growth with IPi, and Pikovskaya broth with phosphate source replaced by NaH_2_PO_4_ for growth with SPi. Data are given as means and standard deviations of biological triplicates. CDW, cell dry weight.

Furthermore, the levels of the compatible solutes trehalose and glycine betaine in supernatants and cell lysates of SBR5 cultivated in SPi or IPi were determined. This was done because, although the levels of osmolality obtained in the supernatants of SPi and IPi cultures were similar (Figure 6A), the internal osmolality of cells was threefold higher than that in SPi cultivation broth (Figure 6B). Approximately 0.01 mM of trehalose was present in SBR5 supernatants at 0 h under both Pi conditions, indicating that this component was present in the media composition (Figure 6C). However, trehalose concentration in the supernatant increased during growth, peaking at 4.9 and 7.8 mM in SPi and IPi, respectively (Figure 6C). SBR5 cells cultivated in IPi medium accumulated trehalose intracellularly from 2 to 8 h of growth, peaking at 0.1 mg g CDW^–1^ at 4 h (Figure 6D). No glycine betaine was detected in the supernatant of SPi cultivation broth, but 24.3 mM of glycine betaine production was observed at 2 h of cultivation in IPi broth (Figure 6E). The glycine betaine concentration in SBR5 supernatants from IPi cultivation dropped completely at 24 h, as this component accumulated intracellularly (Figures 6E,F). The presence of glycine betaine in SBR5 cell lysates was observed from 4 h of growth, being threefold higher at 8 h of growth when those cells were cultivated in IPi in comparison with SPi medium (Figure 6F). Glycine betaine and trehalose were produced and/or accumulated in IPi cultivation broth.

**FIGURE 7:**
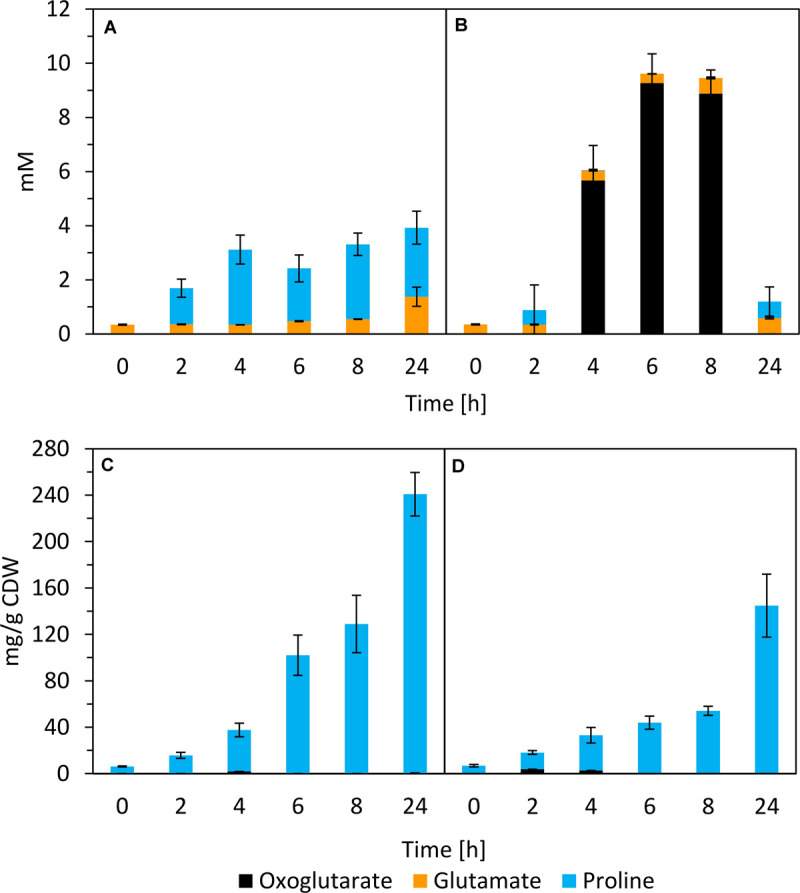
Quantification of oxoglutarate, glutamate, and proline in supernatants **(A,B)** and crude extracts **(C,D)** of *P. sonchi* SBR5 along with its growth in IPi (right) or SPi (left). Pikovskaya broth (Pikovskaya, 1948) was used for growth with IPi, and Pikovskaya broth with phosphate source replaced by NaH_2_PO_4_ for growth with SPi. Data are given as means and standard deviations of biological triplicates.

Glutamate and proline were secreted by SBR5 cells cultivated in IPi (Figures 5B, 7A). The concentration of proline in SBR5 supernatants at 24 h of bacterial growth was fivefold higher in IPi in comparison with SPi (Figures 7A,B). Moreover, the internal proline concentration reached ∼240 mg gCDW^–1^, which is twofold higher than that in SPi (Figures 7C,D). Although ∼9-mM oxoglutarate formation was observed in SPi supernatant (Figure 7B), no oxoglutarate was detected in the supernatant of IPi broth (Figure 7A).

### Thiamine Biosynthesis Confirmation by Flow Cytometry

**FIGURE 8:**
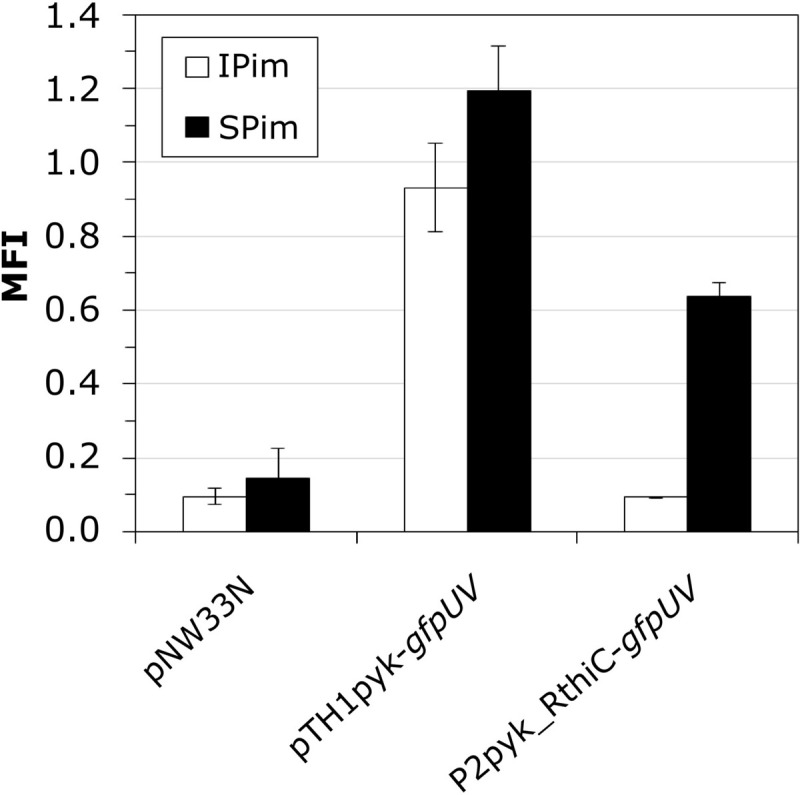
Monitoring intracellular thiamin concentrations by a thiamin-responsive riboswitch fused to GfpUV. *P. sonchi* SBR5 cells carried plasmids either with *gfpUV* gene under control of *pyk* promoter and 5′UTR of the endogenous *thiC* gene (pTH1pyk-*gfpUV*), *pyk* promoter carrying its native 5′UTR (p2pyk_RthiC-*gfpUV*), or the empty vector plasmid pNW33N. *P. sonchi* cells were cultivated in modified IPi or with SPi. MFI (mean fluorescence intensities) are given as means and standard deviations of triplicate cultivations measured by flow cytometry of 20,000 cells. Modified IPi and SPi media were prepared without yeast extract but with addition of biotin (100 μg/ml).

Our RNAseq analysis revealed the upregulation of many thiamine biosynthesis-related genes in SBR5 cultivated in IPi condition (Table 1). Therefore, we have performed a thiamine biosynthesis detection using a thiamine pyrophosphate (TPP)-based biosensor. The strain SBR5(P2pyk_RthiC-*gfpUV*) carries the constitutive promoter of the pyruvate kinase-encoding gene *pyk* of SBR5 with its native 5′ untranslated region (5′UTR) replaced by the 5′UTR of the P. riograndensis_final_150, *thiC* gene coding for phosphomethylpyrimidine synthase (Brito et al., 2017a). The 5′UTR of *thiC* gene contains a TPP riboswitch previously characterized, which “switches off” the expression of *gfpUV* reporter gene in the presence of thiamine (Brito et al., 2017a). The strain SBR5 carrying the plasmid P2pyk-*gfpUV* (Brito et al., 2017b), with original *pyk* 5′UTR and the empty vector pNW33N were used as control. All recombinant strains were cultivated in modified IPi (IPim) or SPi (SPim), both media containing 0.1 mg/L of biotin but lacking yeast extract. Cells were harvested after 6 h, and GfpUV mean fluorescence intensity (MFI) was measured by flow cytometry. As expected, the MFI of empty vector control strain presented GfpUV background signal (Figure 8) in both media. Recombinant SBR5 with native *pyk* promoter driving the expression of *gfpUV* showed elevated GfpUV MFI in both media in comparison with the empty vector control. However, in the presence of *thiC* TPP riboswitch, cells cultivated in IPim “switched off” *gfpUV* expression, being GfpUV MFI levels similar to the empty vector control (Figure 8), indicating thiamine biosynthesis in this condition.

## Discussion

Here, the activity of *P. sonchi* genomovar Riograndensis SBR5 under IPi condition was characterized. Functional analyses were performed based on the currently available genomic and genome-wide transcriptomic databases (Brito et al., 2015, 2017a) and on the gene expression analysis performed in the present study. We aimed to relate some genes of *P. sonchi* SBR5, which were differentially expressed with two distinct P sources (SPi and IPi) to the physiological analysis of PS processes. Our findings revealed that IPi condition (with hydroxyapatite as P source) changed carbon metabolism and vitamin biosynthesis of SBR5 and expressed flagellation genes and activated osmoprotection.

It is a general phenotype of PSB that the PS activity is induced by low levels of exogenous soluble phosphate and inhibited by its high levels (Zeng et al., 2017). The phosphate in SPi condition was not completely utilized (Figure 3B) but supported the growth of *P. sonchi* SBR5 (Figure 3A), indicating that SPi provided a sufficient supply of P for SBR5. However, soluble phosphate was not promptly available for SBR5 at the beginning of growth in IPi medium, containing hydroxyapatite (Figure 3B), lacking the necessary Pi supply for the cells. Altogether, that could explain the increase of ∼1 mM orthophosphate concentration after 24 h in the IPi medium (Figure 3B). This value is similar to values observed for other *Paenibacillus* species: ∼1 mM by *P. polymyxa* and ∼1.2 mM by *P. mucilaginosus* from CaHPO_4_ and phosphorite after 3 and 5 days of incubation, respectively (Hu et al., 2006; Wang et al., 2012). However, the increase in orthophosphate concentration observed in IPi condition represents approximately 3.4 % of hydroxyapatite solubilization rate, which is dramatically inferior to the solubilization rates (up to 100%) reported in fungi (Mendes et al., 2013). It is known that the PS activity of PSB can be repressed by soluble phosphate by a feedback inhibition (Zeng et al., 2017), which is a severe limitation to the extensive application of PSB. However, the molecular mechanism of soluble phosphate regulation on PS activity of PSB remains to be elucidated.

In the past decades, enzymatic processes have been characterized to be responsible for the solubilization of organic phosphate substrates in bacteria. PS promoted by acid phosphatase was observed in *Pseudomonas* sp. (Jorquera et al., 2008), *Burkholderia cepacia* (Rodriguez et al., 2000), *Enterobacter aerogenes*, *Enterobacter cloacae*, *Citrobacter freundi*, *Proteus mirabalis*, and *Serratia marcenscens* (Thaller et al., 1995). Moreover, organic Pi solubilization through phytase activity was observed in *Bacillus subtilis*, *Pseudomonas putida*, and *Pseudomonas mendocina* (Richardson and Hadobas, 1997), and phosphotase activity was detected in *Klebsiella aerogenes* (Ohtake et al., 1996) and *P. fluorescens* (Lidbury et al., 2016). However, although *P. sonchi* SBR5 possesses some of those PS-related enzymes, upregulation of their respective genes was not observed in the differential gene expression analysis under PS conditions (Tables 1, 2). This could be because the phosphate sources utilized in SPi (NaH_2_PO_4_) and IPi (hydroxyapatite) conditions were not from an organic origin. However, an inspection of the complete genome sequence of SBR5 (Brito et al., 2015) allowed the detection of some candidate genes coding for enzymes that promote organic PS and enzymes related to P metabolism, e.g., an alkaline phosphatase (P.riograndensis_final_731) and a phytase (P.riograndensis_final_2549). The Pst phosphate-specific transport system is a major phosphate transport system characterized in *B. subtilis* (Qi et al., 1997). The *pst* operon of *B. subtilis* is composed of *pstS*, *pstC*, *pstA*, *pstB1*, and *pstB2* genes. PstS is a binding protein, PstC and PstA are two integral inner membrane proteins, and PstB1 and PstB2 are ATP-binding proteins (Qi et al., 1997; Atalla and Schumann, 2003). Those genes were found in the genome of *P. sonchi* SBR5 (P.riograndensis_final_3645-3648) and were expressed at low levels in our previous RNAseq study, in which no phosphate depletion treatment was applied (Brito et al., 2017a). Here, the gene *pstS* was downregulated in IPi condition (Table 2) as confirmed by *gfpUV* expression analysis, in which *pstS* promoter-driven expression reduced GfpUV fluorescence in comparison with SPi condition (Figure 4). Our orthophosphate quantification of IPi supernatants revealed P concentrations ∼10 times smaller than the Monod constant depicted in Supplementary Figure 2. This indicates that P is not promptly available for SBR5 cells in the first hours of growth (Figure 3B). Although *pst* P transport genes are stimulated upon P starvation, the underlying regulatory mechanism remains to be studied. Moreover, cultivation of SBR5 in low and high phosphate conditions (LPi and HPi media), together with phosphate quantification and qRT-PCR, revealed that phosphate starvation response of SBR5 could be mediated by a regulatory system. Some bacteria present a global regulatory mechanism of Pi management in a situation of Pi depletion that involves a two-component regulatory system and is named *pho* regulon (Santos-Beneit, 2015). The *pho* regulon is controlled by the two-component PhoP-PhoR signal transduction system (Shi and Hulett, 1999). Homologous genes are present in the genome sequence of *P. sonchi* SBR5 (P.riograndensis_final_2307-2308). Here, qRT-PCR showed that genes involved in the regulation of the *pho* regulon genes *phoU* and *phoH* (Santos-Beneit, 2015) were highly expressed in LPi condition (Figure 2). Phosphate depletion led to the expression of σ^*B*^-mediated general stress response genes in *B. subtilis* (Prágai and Harwood, 2002). *B. subtilis* responds to phosphate starvation stress by regulating genes encoding the phosphate starvation Pho proteins (Prágai et al., 2004). Genes classically associated with their regulation were targeted by qRT-PCR in the present study, being highly expressed by SBR5 cultivated in LPi condition. Among them are *pstS* and *pstB3* and the glycerophosphodiester phosphodiesterase gene *glpQ* (Figure 2), in which the product hydrolyzes deacylated phospholipids to alcohol and glycerol-3-phosphate, which is subsequently utilized in the cell (Jorge et al., 2017a). Finally, the fact that the concentration of orthophosphate in LPi medium dropped approximately 30% at 24 h indicates consumption of the low P available in Pi depletion condition (Figure 1B), which is in accordance with the high expression of high-affinity Pi uptake system *pst* genes (Figure 2).

**FIGURE 9:**
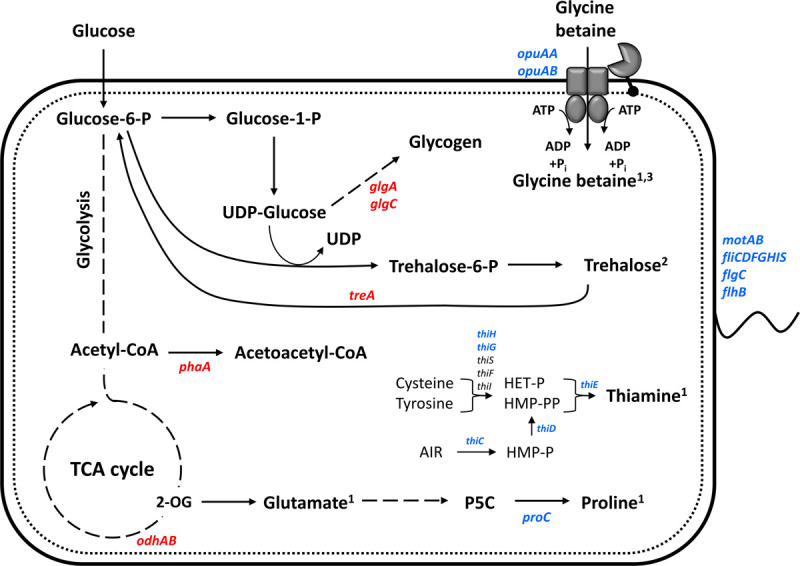
Schematic representation of physiological and gene expression differences between *P. riograndensis* SBR5 grown with IPi and SPi. Genes showing higher RNA levels during growth with IPi as compared with SPi are depicted in blue, whereas those with reduced RNA levels in red. RNAseq together with the physiological analyses of the present study indicates that proline is produced and glycine betaine produced and accumulated, and trehalose is accumulated inside the cell for osmoprotection. Thiamine biosynthesis is activated in insoluble phosphate condition. Continuous lines: one-step processes; dashed lines: processes with more than one-step. ^1^Produced component; ^2^putatively accumulated component; ^3^imported component.

Under IPi condition, several genes involved in thiamine biosynthesis were upregulated (Figure 9). Thiamine is indispensable for the activity of the carbohydrate and branched-chain amino acid metabolic enzymes (Du et al., 2011). TPP is the active form of this vitamin and functions as a cofactor of several important enzymes in carbohydrate and amino acid metabolism (Melnick et al., 2004). Biosynthesis of thiamine has been studied in several groups of PGPR, including *Azotobacter*, *Pseudomonas*, and *Azospirillum* (Palacios et al., 2014). As biotin, thiamine is part of the vitamin B group, which is suggested to improve the plant root colonization by PGPR (Palacios et al., 2014). Thiamine also acts as a cofactor of indolepyruvate decarboxylase, a synthesizing enzyme of principal plant hormone in PGPR indole-3-acetic acid (Schütz et al., 2003). In *E. coli*, thiamine precursors hydroxymethyl-pyrimidine diphosphate and hydroxyethyl-thiazole phosphate are utilized to synthetize TPP (Begley et al., 1999). Thiamine biosynthesis protein ThiC converts aminoimidazole ribotide to hydroxymethyl-pyrimidine phosphate, which is subsequently phosphorylated by the bifunctional hydroxymethyl-pyrimidine phosphate P kinase ThiD to yield hydroxymethyl-pyrimidine pyrophosphate. On the other hand, the thiazole moiety of thiamine is derived from tyrosine, cysteine, and 1-deoxy-D-xylulose-5-phosphate. In a yet unresolved chain of reactions featuring *thiF*, *thiS*, *thiG*, *thiH*, and *thiI* gene products, hydroxyethyl-thiazole phosphate is formed. Hydroxymethyl-pyrimidine pyrophosphate and hydroxyethyl-thiazole phosphate are joined by one enzymatic step mediated by the ThiE protein (Figure 9) (Lünse et al., 2014). In the present study, among the mentioned thiamine biosynthesis genes, only *thiF*, *thiS*, and *thiI* were not detected in RNAseq analysis (Table 1). Moreover, although the production of thiamine by SBR5 was not measured, a TPP riboswitch was used to monitor intracellular TPP. As shown by Brito et al. (2017a), the 5′UTR of the phosphomethylpyrimidine synthase-encoding gene (P.riograndensis_final_150) contains the sequence of a TPP riboswitch that “switches off” gene expression in the presence of 5 μM thiamine. Here, *gfpUV* reporter gene expression was “switched off” in IPim condition, indicating thiamine biosynthesis by *P. sonchi* (Figure 8). These findings suggest that thiamine might be important for SBR5 to cope with low phosphate concentrations during growth in IPi condition.

SBR5 acidifies the IPi medium, whereas it maintains the medium pH near neutral under SPi condition (Supplementary Figure 1), which was expected because acidification typically is the main PS mechanism of PSB (Chen et al., 2006; Marra et al., 2015). The decrease of pH as a mechanism to perform PS has been reported in fungi (Whitelaw, 2000), and it is mostly related to the production of organic acids, as in *Aspergillus* and *Penicillium* (Li et al., 2016). Organic acid release is classically related to PS in bacteria (Illmer and Schinner, 1992). Production of oxalate and malate, which were previously related to PS in *Pseudomonas* (Vyas and Gulati, 2009), was not observed in SBR5. In the PGPR *Burkholderia multivorans*, the expression of genes related to carbon metabolism, including genes encoding for sugar ABC transporters and 2-OGDH, was upregulated when P was depleted in the medium (Zeng et al., 2017). The switch of carbon metabolism pathways is closely related to the depletion of available soluble phosphate (Thomas et al., 2012). Here, downregulation of 2-OGDH genes *odhA* and *odhB* was observed during the growth of SBR5 in IPi condition (Table 2), which was confirmed by reporter gene fusion analysis (Figure 4C). 2-OGDH is a key enzyme that catalyzes the step in TCA cycle in which oxoglutarate is converted to succinyl-CoA (Nguyen et al., 2015). Thereby, it is suggested that the metabolic flux toward the TCA cycle is reduced when SBR5 is cultivated in a medium with IPi, which leads to a general decrease of accumulation of TCA metabolites, e.g., oxoglutarate and succinate (Figures 5A, 7A). Low production of oxoglutarate in contrast to high production of gluconate was also observed in soil bacterial isolates that perform PS (Mardad et al., 2013). Succinate is a product of a step in the TCA cycle that follows the reaction catalyzed by 2-OGDH, and it is also one of the major organic acids present in root exudates of plants in the rhizosphere. Repression of glucose utilization by succinate is termed as succinate-mediated catabolite repression (Bringhurst and Gage, 2002). Divya et al. (2011) showed that succinate and malate individually and as mixtures repressed gluconate production and PS in *Pseudomonas aeruginosa*. It is also a component that represses PS phenotype in *Klebsiella pneumoniae* (Rajput et al., 2013, 2015). Based on that and our findings, the reduction of the metabolic flux toward the TCA cycle might be one strategy utilized by SBR5 to perform PS.

In RNAseq cultivation, the total concentration of analyzed organic acids at the end of SBR5 growth in IPi condition was 15% higher than in SPi (Figure 5A). This is in accordance with our observation regarding the acidification of the growth medium in the IPi condition (Supplementary Figure 1). Gluconate and acetate, known as active agents in PS, were highly produced in IPi condition (Figure 5A). Gluconate is produced by the majority of the PSB, being often the most quantitatively produced organic acid for PS ends (Yi et al., 2008; Vyas and Gulati, 2009; Shahid and Hameed, 2012; Marra et al., 2015). The production of acetate by PSB isolates to perform PS has also been reported (Mardad et al., 2013). The gene encoding the enzyme glucose dehydrogenase is present in the genome of *P. sonchi* SBR5 (P.riograndensis_final_6601). Glucose dehydrogenase oxidizes glucose to convert it to gluconate (Werra et al., 2009). There is a high conservation of this gene in *Paenibacillus* species (Xie et al., 2016). Although gluconate is accumulated in IPi condition, P.riograndensis_final_6601 was slightly transcribed in landscape transcriptome of SBR5 (Brito et al., 2017a) and not detected in our RNAseq analysis. Phosphotransacetylase and acetate kinase are enzymes involved in the reversible interconversion of acetyl-CoA to acetate (Wendisch et al., 1997). The genes P.riograndensis_final_3937 and P.riograndensis_final_4369 (coding for phosphotransacetylase and acetate kinase, respectively) were not differentially expressed, but their transcript abundances were considered intermediate to high in landscape transcriptome analysis (Brito et al., 2017a). In *Sinorhizobium meliloti*, the genes encoding phosphotransacetylase and acetate kinase are induced by phosphate deficiency and controlled by the *pho* regulon (Summers et al., 1999).

The expression of flagellin protein-encoding gene (P.riograndensis_final_6164), FliS (P.riograndensis_final_6161), flagellar capping protein (P.riograndensis_final_6162), hypothetical protein similar to FlaG (P.riograndensis_final_6163), *motAB* genes, FlgC-encoding gene (P.riograndensis_final_4022), flagellar biosynthetic protein FlhB-encoding gene, and genes *fliFGHI* were upregulated under IPi condition (Table 1). Flagella biosynthesis is energy-intensive, and flagella genes are typically expressed from strong promoters. Besides, flagellin proteins are translated from near-consensus RBS (Guttenplan and Kearns, 2013), which is also true for SBR5, where the RBS sequence 5′GGGGAGG assigned to the flagellin gene P.riograndensis_final_6164 is similar to the consensus RBS sequence 5′aGGaGg (Brito et al., 2017a). However, no difference in swimming of SBR5 was detected between SPi and IPi soft agar media (0.2% agar, data not shown). Nevertheless, the acidification of media causes the cells to change the flagella morphology immediately in *E. coli*. When the bacteria were grown at pH 6 or 8, shorter and thinner forms of flagella were produced (Chang et al., 2013). Hence, this indicates that the upregulation of several flagellation-related genes in IPi condition might be due to the change of pH that occurs in this condition.

Another effect observed in our work was the increase in cell osmolality caused by the IPi medium, which led to the change of compatible solutes dynamics in SBR5 cells to induce osmoadaptation (Figure 6). The osmolality of the rhizosphere is likely to exceed that of bulk soil water as a result of the exclusion of salts such as NaCl from root tissue, the exudation of low molecular weight organic acids, and the secretion of mucilage by roots and bacteria (Miller and Wood, 1996). As the activities of roots and rhizobacteria modify the osmolality of the rhizosphere, osmoadaptive mechanisms will likely influence both the survival of rhizosphere bacteria and their interactions with plant roots. The study of osmoadaptation by rhizosphere bacteria has been limited to a small number of genera. In most cases, these bacteria have been examined because of interest in their interactions with plants rather than their degree of salt tolerance (Miller and Wood, 1996). Soil variables that influence the osmotic properties of the rhizosphere include its clay content and texture, its water content, and the nature and concentration of solutes in the soil water (Gregory, 1988). The osmolality of typical soil water has been estimated as less than 50 mOsm kg^–1^ (Gregory, 1988). Here, the cultivation of SBR5 in IPi condition led to an osmolality as high as 600 mOsm kg^–1^ (Figure 6B). This could be due to the increase of organic acid excretion in this condition, which was 15% higher than in SPi (Figure 5A). The increase in osmolality caused SBR5 cells to use osmoregulatory mechanisms such as production, uptake, and accumulation of compatible solutes (Figure 9). Proline and glycine betaine are among the principal compatible solutes accumulated as an osmotic response in bacteria (Sleator and Hill, 2002). The accumulation of the osmoprotectant glycine betaine from exogenous sources provides a high degree of osmotic tolerance to *B. subtilis* (Kappes et al., 1996). Proline was secreted by SBR5 to higher concentrations under IPi as compared with SPi condition (Figures 5B, 7A). Accordingly, there was upregulation of the gene P.riograndensis_final_4130 (*proC*) encoding pyrroline-5-carboxylate reductase (Table 1), which is the enzyme catalyzing the last step in the proline production pathway in *Corynebacterium glutamicum* (Jensen et al., 2015). Another evidence is the downregulation of the 2-OGDH complex genes *odhAB*, which could have caused a change in the metabolic flux from the TCA cycle intermediate 2-oxoglutarate to proline (Figure 9). The metabolic pull of oxoglutarate toward glutamate and proline biosynthesis may explain the reduced oxoglutarate concentration and the increased glutamate and proline concentrations in supernatants of IPi cultures in comparison with SPi (Figures 7A,B). Three lines of evidence showed intracellular accumulation of glycine betaine under IPi condition: *opuAA* and *opuAB* RNA levels were increased (Table 1), GfpUV reporter gene expression driven by *opuAA* promoter increased (Figures 4D,E), as did the intracellular glycine betaine concentration (Figure 6F). Lastly, trehalose was accumulated in SBR5 cells in IPi condition (Figure 6D). Accordingly, the reduced expression of *treA*, *glgA*, and *glgC* genes was observed. Trehalose is degraded by trehalase TreA, whereas GlgC and GlgA synthesize glycogen, a pathway competing for UDP-glucose with trehalose synthesis (Purvis et al., 2005). Thus, the changed medium and inner cell osmolality under IPi condition were counteracted by the secretion of proline and glutamate and the accumulation of glycine betaine and trehalose.

Taken together, our differential gene expression study revealed an extraordinarily complex transcriptional response of *P. sonchi* SBR5 to two distinct P sources, soluble and insoluble. On the one hand, *P. sonchi* SBR5 changes its carbon metabolism characteristics in the presence of insoluble phosphate by the reduction of the metabolic flux toward the TCA cycle. On the other hand, a drastic change in cell osmolality was detected, triggering osmoprotection mechanisms (Figure 9). We showed that the production of organic acids might be the most important strategy utilized by SBR5 to perform PS. The findings of our study will help us understand the mechanisms of insoluble phosphate-derived gene regulation and physiological activities by *P. sonchi* SBR5, providing the first step in the elucidation of the PS process in this organism, which could further improve the scope of its application as a crop inoculant.

## Data Availability Statement

The datasets presented in this study can be found in online repositories. The names of the repository/repositories and accession number(s) can be found below: https://www.ncbi.nlm.nih.gov/geo/, GSE154303.

## Author Contributions

LB, MGL, and LS carried out the experimental procedures. LB analyzed the data of the present study. LB prepared a draft of the manuscript. LB, LP, and VW finalized the manuscript. VW coordinated the study. All the authors read and approved the manuscript.

## Conflict of Interest

The authors declare that the research was conducted in the absence of any commercial or financial relationships that could be construed as a potential conflict of interest.
