# Supplementary material for: Inorganic Phosphate Solubilization by Rhizosphere Bacterium Paenibacillus sonchi: Gene Expression and Physiological Functions
Source: Front Microbiol. 2020 Dec 14;11:588605. doi: 10.3389/fmicb.2020.588605 (PMC7793946; doi:10.3389/fmicb.2020.588605)
Supplement: Supplementary file 1 [file Data_Sheet_1.docx]

**SUPPLEMENTARY MATERIAL**

**Inorganic phosphate solubilization by rhizosphere bacterium** ***Paenibacillus sonchi*: gene expression and physiological functions**

Luciana F. Brito^1#^, Marina Gil López^1#^, Lucas Straube^1^, Luciane M. P. Passaglia^2^ & Volker F. Wendisch^1*^

^1^Bielefeld University, Genetics of Prokaryotes, Faculty of Biology & Center for Biotechnology (CeBiTec), Universitaetsstraße 25, 33615 Bielefeld, Germany

^2^UFRGS, Universidade Federal do Rio Grande do Sul, Department of Genetics, Porto Alegre, Brazil

^#^Current address: NTNU, Norwegian University of Science and Technology, Department of Biotechnology and Food Science, Trondheim, Norway

^*^Corresponding author: Bielefeld University, Genetics of Prokaryotes, Faculty of Biology & Center for Biotechnology (CeBiTec), Universitaetsstraße 25, 33615 Bielefeld, Germany; [volker.wendisch@uni-bielefeld.de](mailto:volker.wendisch@uni-bielefeld.de)

**Table S1. Strains, plasmid DNA and oligonucleotides sequences used in the present study for molecular cloning, plasmid constructions and strain development.** Overlaps are in bold.

| **Bacterial Strain** | **Characteristics** | **Source** |
| --- | --- | --- |
| *Escherichia coli* | Wild-type, DH5α | Hanahan (1983) |
| *P. sonchi* | Wild-type, SBR5 | UFRGS |
| *P. sonchi* | SBR5(P2pyk-*gfpUV*), CmR, KmR; pNW33N-based, *pyk* gene promoter (amplified from SBR5 genome) upstream *gfpUV* gene | Brito et al., (2017) |
| *P. sonchi* | SBR5(P2pyk_RthiC-*gfpUV*), CmR, KmR; pNW33N-based, *pyk* gene promoter with 5'UTR of *thiC* gene (both amplified from SBR5 genome) upstream *gfpUV* gene | Brito et al., (2017) |
| **Plasmid DNA** | **Characteristics** | **Source** |
| pNW33NKan (WT) | CmR, KmR; pNW33N derivative | Irla et al., (2016) |
| P2odHA-*gfpUV* | CmR, KmR; pNW33NKan plasmid with *odhA* gene promoter (amplified from SBR5 genome) upstream *gfpUV* gene | This work |
| P2pstS-*gfpUV* | CmR, KmR; pNW33NKan plasmid with *pstS* gene promoter (amplified from SBR5 genome) upstream *gfpUV* gene | This work |
| P2opuAA-*gfpUV* | CmR, KmR; pNW33NKan plasmid with *opuAA* gene promoter (amplified from SBR5 genome) upstream *gfpUV* gene | This work |
| P2pstS-*gfpUV*_opuAA-*crimson* | CmR, KmR; pNW33NKan plasmid with *pstS* gene promoter (amplified from SBR5 genome) upstream *gfpUV* gene and *opuAA* gene promoter (amplified from SBR5T genome) upstream *crimson* gene | This work |
| **Oligonucleotide** | **Sequence (5'-3')** | **Comment** |
| odhAfw | **gaattcgagctcggtacccggg**aatacacccattctaaatccagatcacccg | Amplifies *odhA* promoter; overlaps with pNW33N BamHI site |
| odhArv | **gtgaaaagttcttctcctttactcat**gaatacatcctcctaacggatgaactcc | Amplifies *odhA* promoter; overlaps with *gfpUV* |
| gfp1fw | **ggagttcatccgttaggaggatgtattc**atgagtaaaggagaagaacttttcac | Amplifies *gfpUV*; overlaps with *odhA* promoter |
| gfp1rv | **ctggcccgatgactctagaggatc**ttatttgtagagctcatccatgcc | Amplifies *gfpUV*; overlaps with pNW33N BamHI site |
| pstSfw | **gaattcgagctcggtacccggg**tatatttttcaattagcactacaatttg | Amplifies *pstS* promoter; overlaps with pNW33N BamHI site |
| pstSrv | **gtgaaaagttcttctcctttactcat**ttttttgtctcctcctgagtgttttg | Amplifies *pstS* promoter; overlaps with *gfpUV* |
| gfp2fw | **caaaacactcaggaggagacaaaaaa**atgagtaaaggagaagaacttttcac | Amplifies *gfpUV*; overlaps with *pstS* promoter |
| gfp2rv | **ctggcccgatgactctagaggatc**ttatttgtagagctcatccatgcc | Amplifies *gfpUV*; overlaps with pNW33N BamHI site |
| opuAA1fw | **gaattcgagctcggtacccggg**cgctcccgggctttgctaattctctcaac | Amplifies *opuAA* promoter; overlaps with pNW33N BamHI site |
| opuAA1rv | **gtgaaaagttcttctcctttactcat**ggtgacctccttcttccgtatagc | Amplifies *opuAA* promoter; overlaps with *gfpUV* |
| gfp3fw | **gctatacggaagaaggaggtcacc**atgagtaaaggagaagaacttttcac | Amplifies *gfpUV*; overlaps with *pstS* promoter |
| gfp3rv | **Ctggcccgatgactctagaggatc**ttatttgtagagctcatccatgcc | Amplifies *gfpUV*; overlaps with pNW33N BamHI site |
| opuAA2fw | **ttatttgtagagctcatccatgcc**cgctcccgggctttgctaattctctcaac | Amplifies *opuAA* promoter; overlaps with *gfpUV* end of P2pstS-*gfpUV* backbone |
| opuAA2rv | **gtgaaaagttcttctcctttactcat**ggtgacctccttcttccgtatagc | Amplifies *opuAA* promoter; overlaps with *crimson* |
| crimfw | **gctatacggaagaaggaggtcacc**atggatagcactgagaacgtcatcaag | Amplifies *crimson*; overlaps with *opuAA* promoter |
| Crimrv | **Ctggcccgatgactctagaggatc**ctactggaacaggtggtggc | Amplifies *crimson*; overlaps with pNW33N BamHI site |
| colpNW33fw | Cccgttctctctgattgtgaaattg | Oligonucleotides utilized for colony PCR |
| colpNW33gfprv | Gtagagctcatccatgccatgtgtaatccc | Oligonucleotides utilized for colony PCR |

**Table S2. Oligonucleotide sequences (5’-3’) used for amplification of gene fragments of *P. sonchi* SBR5 in qRT-PCR.**

| **Gene identity** | **Forward** | **Reverse** | **Gene product length [bp]** |
| --- | --- | --- | --- |
| P.riograndensis_final_1896 | gctgagctgatgggtgaatg | tatccgggatgctctccttg | 229 |
| P.riograndensis_final_1897 | tcgtctatgcgcttggatcg | aagtgaacgtccgctgatcg | 179 |
| P.riograndensis_final_1898 | ccgccagcaaagcctatctc | gctacatcatcgcccagctc | 154 |
| *yusV* | gcatcaaggctggctgaagg | agataggtggtcggctcgtc | 198 |
| P.riograndensis_final_1900 | agcgggtcaaggccagaatc | ccgccacaagggttctttgc | 243 |
| *odhB* | tgccgtgtctgccaaggaag | atgccgagaatgccgacctg | 231 |
| *odhA* | ggcacaccgatccggttaag | cgggtgcgaacacgttgtag | 208 |
| P.riograndensis_final_5412 | atcggatgccagcattgtag | aatgtagccgatggcgttag | 181 |
| P.riograndensis_final_5413 | gaactgaacgacagcaaagg | ccgtctgcattccaatgaag | 242 |
| P.riograndensis_final_5640 | gtgtggcaaagtccctgaac | caaacatccagtgcggtgtc | 210 |
| P.riograndensis_final_5641 | cctattggatcggcaagtgg | ccggtgtcactgtattctgg | 198 |
| *opuAA* | ccgaatatggccttgaagtg | cactgaaagcctcatccatc | 201 |
| P.riograndensis_final_6162 | gcggtgtatgacactgcttc | tagcgcccttcaaggagatg | 214 |
| P.riograndensis_final_6163 | tgctgcagtacagggagttg | tccttgcactgcccgaattg | 157 |
| P.riograndensis_final_6164 | cgagcaagcacaacgtaatg | tatttgcacccgctgtgatg | 245 |
| *pstB3* | aagtgggcatggtgtttcag | gcacgggcaatacaaagacg | 226 |
| *phoU* | cctgcgccgcattattgttg | cggtattctcgtccagatag | 200 |
| *pstS* | atatcgggaactccgatgtg | taccgccaacctctttccag | 204 |
| *phoH* | aagcgggacatcgtcttcgg | tagagcggacggagatatgg | 200 |
| *glpQ* | ggctgcggctgaacattgag | gatcaccggatctggagtcg | 201 |
| Reference control (16S)* | cacgtgtagcggtgaaatgc | acttcggcaccaagggtatc | 184 |

* Oligonucleotide sequences designed by (Sperb et al., 2016).

**Table S3.** Sequencing and mapping features of cDNA libraries of *P. sonchi* SBR5.

|  | **IPi** | **SPi** |
| --- | --- | --- |
| Total reads | 2,773,600 | 2,729,614 |
| Reads after trimming | 2,665,163 | 2,631,198 |
| Mapped reads | 2,611,860 | 2,596,992 |


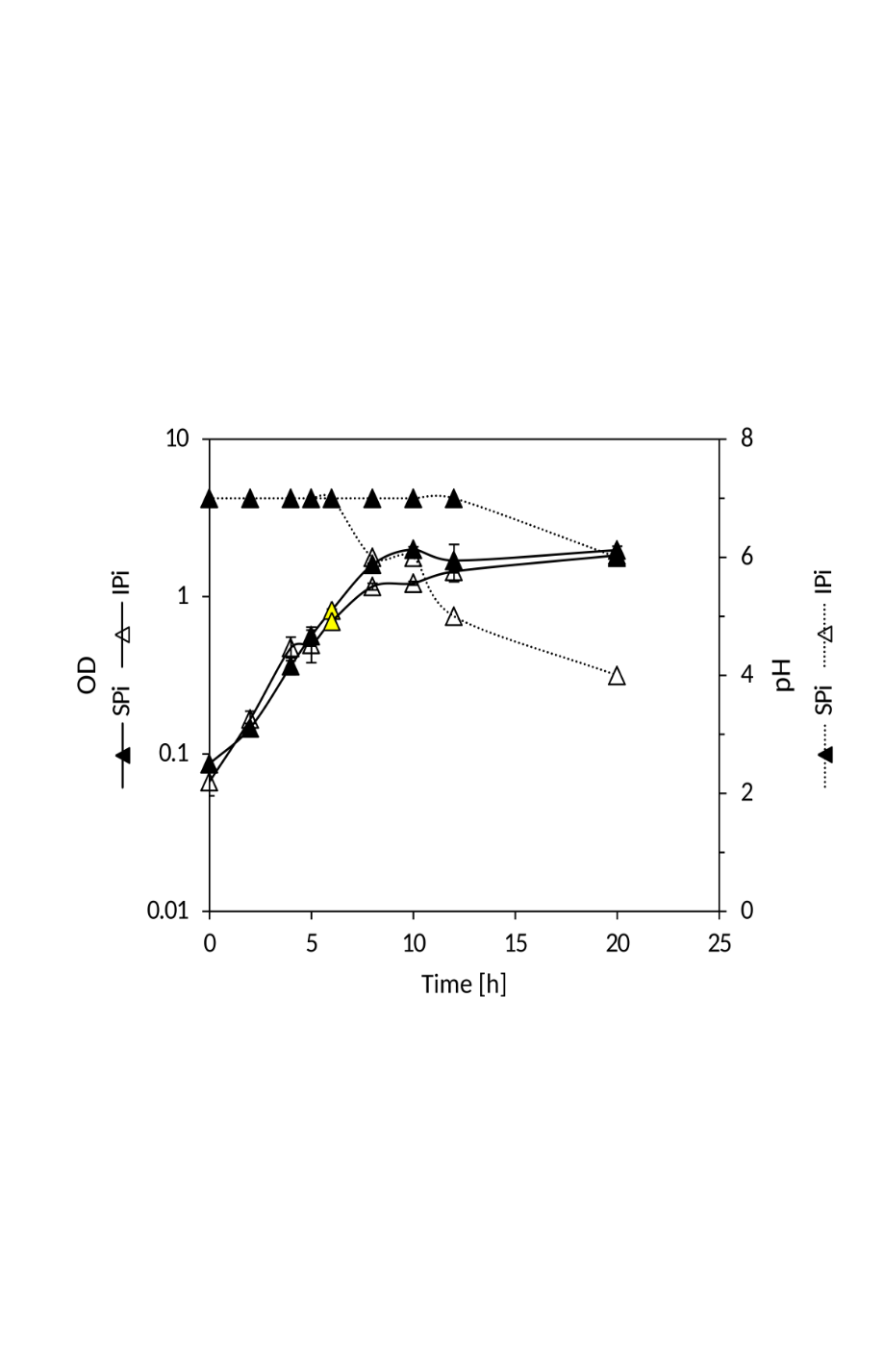


**Figure S1.** **Growth (optical density at 600 nm- OD) and pH values in culture broth during cultivation of *P. sonchi* SBR5 with IPi or SPi.** Pikovskaya broth (Pikovskaya, 1948) was used for growth with IPi, and Pikovskaya broth with phosphate source replaced by NaH_2_PO_4_ for growth with SPI. Data are given as means and standard deviations of biological triplicates. Yellow symbols indicate sampling for RNA isolation.


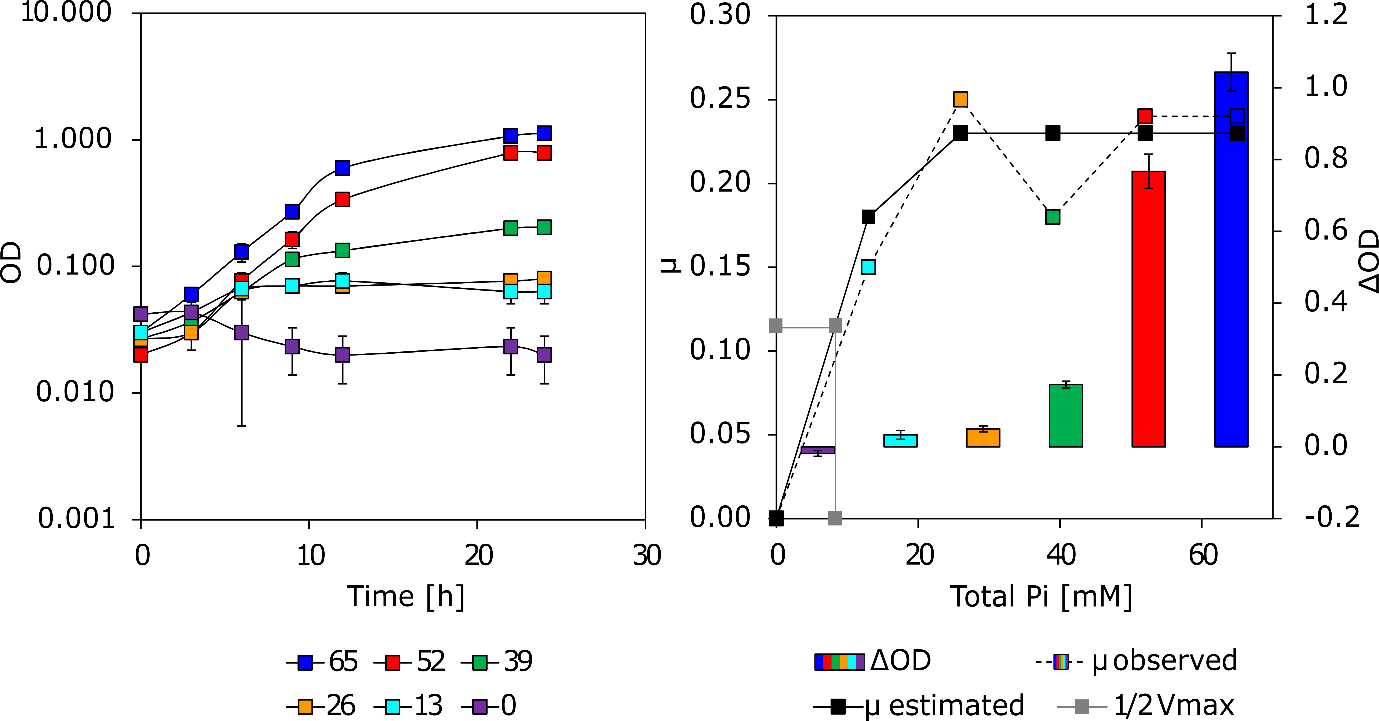


**Figure S2. Growth of *P. sonchi* in CGXII medium with six different concentrations (0, 13, 26, 39, 52 or 65 mM) of inorganic phosphate (left chart) and growth rates and final biomass concentrations (delta optical density at 600 nm- ΔOD) as function of the concentration of inorganic phosphate (right chart).** Data given as means and standard deviations of technical triplicates.

**
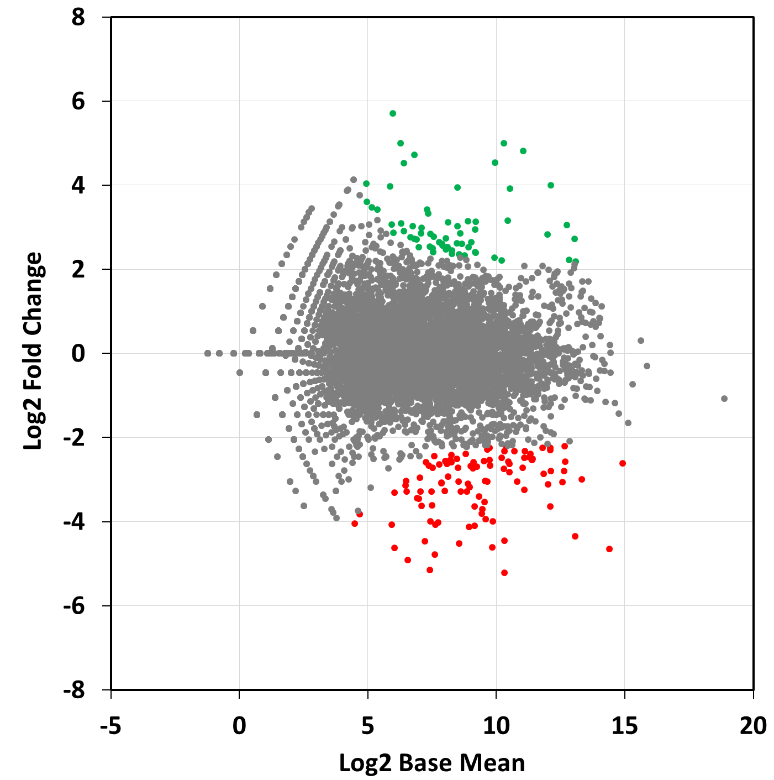
**

**Figure S3. Mean average plot of *P. sonchi* differential gene expression between cultivation in IPi and SPi.** Dots represent significantly upregulated (green) and downregulated (red) genes (*p* < 0.05) or not differentially expressed genes (grey).


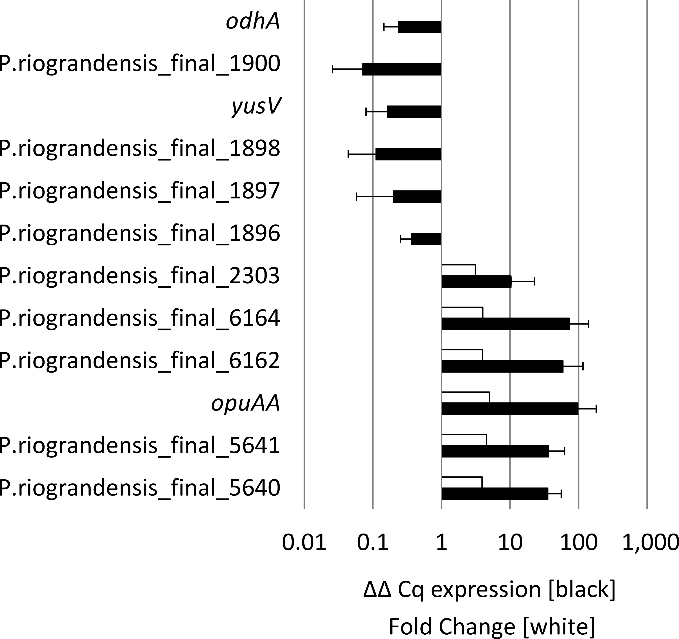


**Figure S4. Comparison of gene expression analysis by RNAseq (white columns) and qRT-PCR (black columns).** Mean ΔΔCq expression determined by qRT-PCR is given as means and standard deviations of triplicate cultivations. RNAseq data were taken from Tables 1 and 2 (fold change in gene expression of *P.sonchi* for growth with IPi as compared to SPi).

**References**

Pikovskaya, R.I. (1948). Mobilization of phosphorus in soil in connection with the vital activity of some microbial species. *Microbiologiya* 17**,** 362-370.

Sperb, E.R., Tadra-Sfeir, M.Z., Sperotto, R.A., Fernandes G.C., Pedrosa, F.O., De Souza, E.M., and Passaglia, L.M. (2016). Iron deficiency resistance mechanisms enlightened by gene expression analysis in *Paenibacillus riograndensis* SBR5. *Res Microbiol* 167**,** 501-509.
